# Source and Delivery of Nutrients to Receiving Waters in the Northeastern and Mid-Atlantic Regions of the United States^1^

**DOI:** 10.1111/j.1752-1688.2011.00582.x

**Published:** 2011-10

**Authors:** Richard B Moore, Craig M Johnston, Richard A Smith, Bryan Milstead

**Keywords:** nutrients, transport and fate, stochastic models, nitrogen, phosphorus, SPARROW

## Abstract

**Abstract:**

This study investigates nutrient sources and transport to receiving waters, in order to provide spatially detailed information to aid water-resources managers concerned with eutrophication and nutrient management strategies. SPAtially Referenced Regressions On Watershed attributes (SPARROW) nutrient models were developed for the Northeastern and Mid-Atlantic (NE US) regions of the United States to represent source conditions for the year 2002. The model developed to examine the source and delivery of nitrogen to the estuaries of nine large rivers along the NE US Seaboard indicated that agricultural sources contribute the largest percentage (37%) of the total nitrogen load delivered to the estuaries. Point sources account for 28% while atmospheric deposition accounts for 20%. A second SPARROW model was used to examine the sources and delivery of phosphorus to lakes and reservoirs throughout the NE US. The greatest attenuation of phosphorus occurred in lakes that were large relative to the size of their watershed. Model results show that, within the NE US, aquatic decay of nutrients is quite limited on an annual basis and that we especially cannot rely on natural attenuation to remove nutrients within the larger rivers nor within lakes with large watersheds relative to the size of the lake.

## Introduction

Elevated concentrations of nutrients (nitrogen and phosphorus) in rivers and lakes throughout the United States frequently result in water-resource impairments (USEPA, 2000a,b;). In the Northeastern and Mid-Atlantic (NE US) regions of the United States, elevated nitrogen concentrations are a common cause of eutrophication of coastal waters (National Research Council, 2000; USEPA, 2000b, 2008) and elevated concentrations of phosphorus are a common cause of eutrophication in freshwater rivers and lakes. Eutrophic waters often exhibit dense growths of algae or other nuisance aquatic plants, depressed levels of dissolved oxygen, loss of fish and submerged aquatic vegetation, and foul odors. In 2000, many lakes in the NE US regions were classified by state and federal agencies as eutrophic, largely due to excess amounts of phosphorus (USEPA, 2000b).

To provide information to support the management of surface waters in the NE US, the U.S. Geological Survey (USGS) developed SPAtially Referenced Regressions On Watershed attributes (SPARROW) nitrogen and phosphorus models representing a 2002 time frame. SPARROW models can be used by water-resources managers as tools in water-quality assessment and management activities such as studies and determinations of total maximum daily loads (TMDLs), nutrient-criteria development, and determination of nutrient loadings to coastal and inland water bodies. SPARROW models can be used to explain spatial patterns in monitored stream-water quality in relation to human activities and natural processes.

The SPARROW modeling approach originally was developed and applied at the national scale to assess nutrient-source contributions, transport, and water-quality conditions for the base year 1987 (Smith *et al.*, 1997). A national model has since been developed to simulate nitrogen and phosphorus loading for the year 1992 (Alexander *et al.*, 2008), and models have been developed for specific drainages within the NE US. These regional models include nutrient models for the Chesapeake Bay watershed (Preston and Brakebill, 1999), the Delaware River watershed (Mary M. Chepiga, USGS, written communication, 2006), and the New England region (Moore *et al.*, 2004).

Modeling results can help in a variety of management decisions, including those related to contaminant reduction and protection strategies across broad regions, and decisions about future monitoring and assessment of streams that are highly vulnerable to environmental degradation. Specifically, for estuaries, the transport of nitrogen from freshwaters to those estuaries has given rise to environmental concerns, such as eutrophication and depletion of oxygen. SPARROW models provide estimates of mean-annual nutrient loading and source allocations to coastal waters. This information can help identify the relative contributions of different river basins to nutrient loads, the sources of the loads, and where potential eutrophication may occur.

Eutrophication of coastal waters from excessive nitrogen loadings within the NE US regions occurs in estuary systems such as Chesapeake Bay, Long Island Sound, and Narragansett Bay. Eutrophication is more apt to occur where there is restricted or delayed mixing and exchange of river and ocean waters. Estuaries with small tidal ranges generally have less mixing and are more prone to eutrophication than those with large ranges in tide; examples include Chesapeake Bay and Long Island Sound (USEPA, 2008). Large annual nitrogen loads from the Susquehanna, Potomac, and the James rivers make Chesapeake Bay especially vulnerable to nitrogen enrichment and eutrophication. Similarly, large annual nitrogen loads from the Connecticut, Providence, and the Housatonic rivers, combined with small tidal ranges and limited mixing, make Long Island Sound especially vulnerable to nitrogen enrichment, eutrophication, and hypoxia (USEPA, 2008).

In some estuaries with large human populations in their watersheds, point sources or wastewater treatment plants are the largest source supplying much of the nitrogen to the estuaries [such as the James (Brakebill and Preston, 2004) and the Merrimack (Moore *et al.*, 2004)]. In other cases, agricultural sources dominate [such as the Potomac and Susquehanna (Brakebill and Preston, 2004)], and in still other cases, atmospheric deposition of nitrogen is the largest single source of nitrogen to estuaries. Some studies have identified atmospheric deposition as the largest single source of nitrogen in the NE US regions (Howarth *et al.*, 1996; Jaworksi *et al*., 1997; Boyer *et al.*, 2002). For New England, an earlier SPARROW model by Moore *et al.* (2004) indicated that 50% of the nitrogen loads delivered to streams were from atmospheric deposition. However, because of instream nitrogen attenuation, especially in the small streams, the share of the load actually delivered to estuaries is less.

The delivery of phosphorus to lakes is important because (1) phosphorus is typically the limiting nutrient for aquatic plant growth in freshwater lakes and ponds (Schindler *et al.*, 2008); and (2) phosphorus accumulates in lake sediments. Unlike nitrogen, for which the primary mechanism of loss is to the atmosphere (i.e., through denitrification), the loss of phosphorus is due to its accumulation in biota and ultimately in sediments. SPARROW models can be useful tools in identifying lakes and embayments that have high concentrations of phosphorus and thus could be vulnerable to eutrophication.

For the purpose of understanding the impact of various nutrient sources and delivery factors, the nitrogen and phosphorus models presented here provide detailed spatial assessments of the region. This is accomplished by using the National Hydrography Dataset Plus (NHDPlus) network to expand the detailed SPARROW model that already existed for New England to include all of the NE US drainages to the U.S. Atlantic Seaboard. The models represent a recent time frame (2002) and appreciably expand the set of monitoring data used to estimate the models. More detailed agricultural (crop-based) sources, for both nitrogen and phosphorus, as well as the wet deposition form of nitrogen (nitrate and ammonium) are incorporated into the models. Also included are delivery factors that are unique or more detailed than those used in previous SPARROW models. All of these advances improve the accuracy of SPARROW models for the region and improve the utility of the model for management applications for both inland and coastal receiving waters.

The objectives of this paper are to (1) document the development of SPARROW nutrient models for the Northeast and Mid-Atlantic regions; (2) describe the application of the nitrogen SPARROW model to investigate the influence of atmospheric deposition and other sources on stream loading and delivery of nitrogen to estuaries; and (3) describe the application of the phosphorus model to investigate phosphorus delivery and loadings to large lakes and reservoirs. The paper also includes three supplementary sections (Supporting Information): (A) nitrogen model coefficients; (B) nitrogen model results by major river – the relative role of atmospheric deposition; and (C) phosphorus model coefficients.

## Methods

**FIGURE 1:**
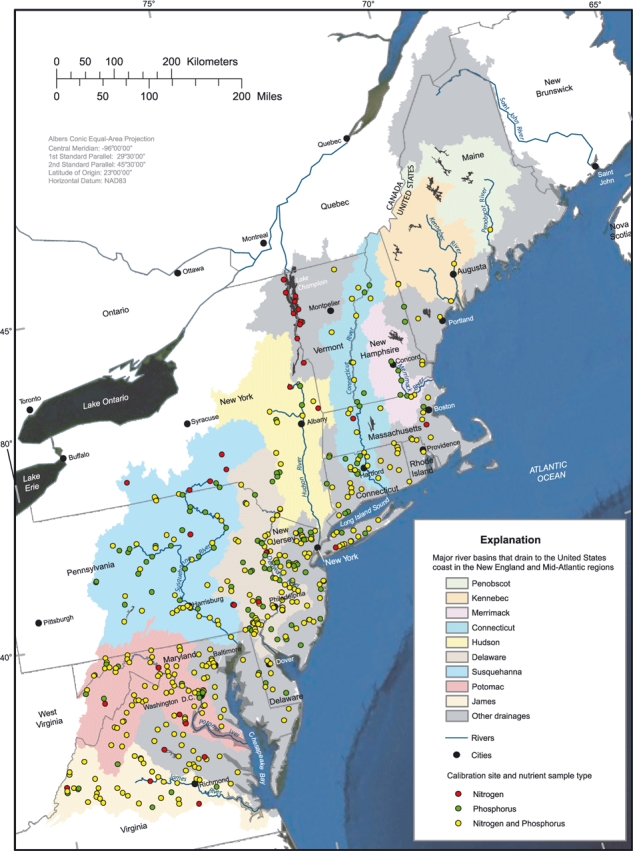
Map of Northeastern and Mid-Atlantic Regions Study Area, Major Watersheds Within the Study Area, and Nutrient Load Calibration Points.

The SPARROW models described in this paper apply to nutrient sources and delivery to waters of the NE US regions of the United States for conditions in 2002, and include all of the watersheds draining to the Atlantic coast from Virginia to Maine, plus drainage to Lake Champlain and Canada (Figure 1). Major NE US watersheds, draining directly to the Atlantic coast, include those of the James, Potomac, Susquehanna, Delaware, Hudson, Connecticut, Merrimack, Kennebec, and Penobscot rivers (Figure 1). Earlier models, for parts or all of the NE US, simulated conditions in the 1980s and early 1990s.

The mathematical form of the SPARROW model is that of a nonlinear-least-squares (NLLS) regression model in which the loads of a nutrient (or other constituent) are weighted by estimates of loss of that nutrient due to land-to-water delivery and instream processing (Smith *et al.*, 1997). The nutrient load at sampled locations is the dependent variable in the model, while source, land-to-water delivery, and instream loss terms are the explanatory variables. Further information about the SPARROW modeling technique and its mathematical form can be found in Preston *et al.* (2009) and Schwarz *et al.* (2006). SPARROW models are designed to simulate the behavior of individual chemical or organic constituents of water; thus separate models were developed for nitrogen and phosphorus. The dependent variable in the model is either mean-annual total nitrogen or total phosphorus load computed for 2002 water-quality conditions, and average annual streamflow conditions (detrended to 2002 if necessary). (Note: throughout this article the terms nitrogen and phosphorous refer to total nitrogen and total phosphorus unless otherwise qualified.) An average streamflow hydrograph was used in the development of the dependent variable in order to eliminate the noise in the dependent variable caused by year-to-year fluctuations in streamflow. The SPARROW model is thus calibrated to, and, in turn, predicts, nutrient loads that represent water-quality source conditions for the specific year of estimation and for longer-term average annual streamflow conditions.

For the Northeast and Mid-Atlantic SPARROW models, a base year of 2002 was selected for estimation. This year was selected to represent more recent conditions than previous SPARROW models and to maximize the amount of nutrient load and explanatory variable datasets that are available for analysis. Several nationwide database development and modeling efforts were necessary to create models consistent with 2002 conditions. These include the compilation of nutrient load monitoring data and explanatory variable data such as that for atmospheric deposition of nitrogen, commercial fertilizer applications to agricultural land, animal-manure production, point-source discharges, population density, and land cover (urban, agricultural, and forested) (Wieczorek and Lamotte, 2011).

The National Water-Quality Assessment Program (NAWQA) of the USGS has developed SPARROW models to assess nutrient conditions in six large regions across the nation for the base year 2002. In addition to the Northeast and Mid-Atlantic model presented here, models have been developed for: the South Atlantic-Gulf and Tennessee basins (Hoos and McMahon, 2009; García *et al.*, this issue); the Great Lakes, Ohio, Upper Mississippi, and Souris-Red-Rainy basins (Robertson and Saad, this issue); the Missouri (Brown *et al.*, this issue); the Lower Mississippi, Arkansas-White-Red, and Texas-Gulf basins (Rebich *et al.*, this issue); and the Pacific Northwest basins (Wise and Johnson, this issue).

### Development of Stream Network

NHDPlus (1:100,000 scale) (U.S. Environmental Agency and USGS, 2006, accessed at http://www.horizon-systems.com/nhdplus/) is the digital representation of the stream network used for the NE US SPARROW models. More than 193,000 NHDPlus flowlines (stream segments and coastal shorelines) within the NE US, complete with incremental watersheds or catchments, with an average size of 2.3 km^2^, were used as the basis for the SPARROW models. By contrast, only 4,944 stream segments and associated catchments, with an average incremental watershed of 90.5 km^2^, are in the Reach File 1 (RF1) network for the NE US. Improvements to the NHDPlus dataset were implemented to develop a stream network for the NE US SPARROW models. Those improvements include (1) making corrections to ensure that all stream reaches are connected appropriately, (2) incorporating major interbasin transfers of water, and (3) adding informative attributes to the streams and their drainages.

NHDPlus is built upon the 1:100,000 scale NHD. NHDPlus includes a stream network, based on the medium resolution NHD (1:100,000 scale) which is described by Simley and Carswell (2009), and “value-added attributes” (VAAs). NHDPlus also includes catchments derived using a digital elevation model that had been modified to ensure drainage to the mapped locations of the streams and away from mapped watershed boundaries. This drainage enforcement technique was first broadly applied for New England SPARROW nutrient models (Moore *et al.*, 2004). This technique involves enforcing the 1:100,000-scale NHD stream network by modifying a grid of elevations from the National Elevation Dataset (NED) and creating artificial trenches along streams. A description of the NED is provided by Gesch *et al.* (2009). The technique also uses the Watershed Boundary Dataset (WBD) (U.S. Department of Agriculture, Natural Resources Conservation Service, 2004) where available, to enforce hydrologic divides (Johnston *et al.*, 2009). NHDPlus data can be accessed through the U.S. Environmental Agency (USEPA) Waters website (http://www.epa.gov/waters/).

NHDPlus includes many features that are useful for SPARROW modeling. Catchment boundaries are necessary in SPARROW modeling to relate watershed characteristics to stream reaches. NHDPlus includes tabular information associated with each reach and catchment and VAAs that are necessary for SPARROW modeling. Other useful NHDPlus VAAs include catchment temperature and precipitation values as well as Strahler Stream Order (Strahler, 1957). In addition, USEPA and USGS have linked numerous water-quality databases to the underlying NHD by assigning NHD stream (Reach) addresses to these entities. Datasets that have been linked include streamflow-gaging stations, water-quality monitoring sites, and impaired waters. The USGS gaging stations and associated data that were used to validate the NHDPlus streamflows and velocity estimates are included with the NHDPlus data.

### Estimating Stream Loads and Selecting Explanatory Variables

Annual stream loads of nutrients (nitrogen or phosphorus) are the dependent variables in the SPARROW models. Loads were estimated on the basis of federal, state, and local water-quality monitoring data (Saad *et al.*, this issue). If the nutrient concentration data were collected by multiple agencies along the same stream reach, then the data were combined and used as if they were all from one site. The data were screened to identify sites (flowlines) with at least 25 samples, at least two years of record, and sites at which samples had been collected within a few years of the SPARROW model year as described in Saad *et al*. (this issue). The resulting set of data, comprising 363 nitrogen and 457 phosphorus measurement sites (Figure 1), represents a wide range of drainage areas. Watersheds for the nitrogen model measurement sites range from 70,132 to 5.7 km^2^, with a median value of 412 km^2^, an upper quartile of 1,492 km^2^, and a lower quartile of 141 km^2^. Watersheds for the phosphorus model measurement sites are similar and range from 70,132 to 5.7 km^2^, with a median value of 434 km^2^, an upper quartile of 1,492 km^2^, and a lower quartile of 132 km^2^.

Explanatory data are used to describe nutrient sources and potential processes of attenuation and ultimately to estimate stream loads. SPARROW models require spatially defined information on specific or potential point and nonpoint sources of nitrogen and phosphorus. All sources evaluated in the models were georeferenced and assigned to the appropriate NHDPlus flowlines or catchments (Maupin and Ivahnenko, this issue; Wieczorek and Lamotte, 2011). Municipal and industrial wastewater discharges of nitrogen or phosphorus input to the models are based on a USEPA permitted wastewater-discharge dataset that was developed on the basis of the methods used by McMahon *et al.* (2007) and described in Maupin and Ivahnenko (this issue).

**FIGURE 2:**
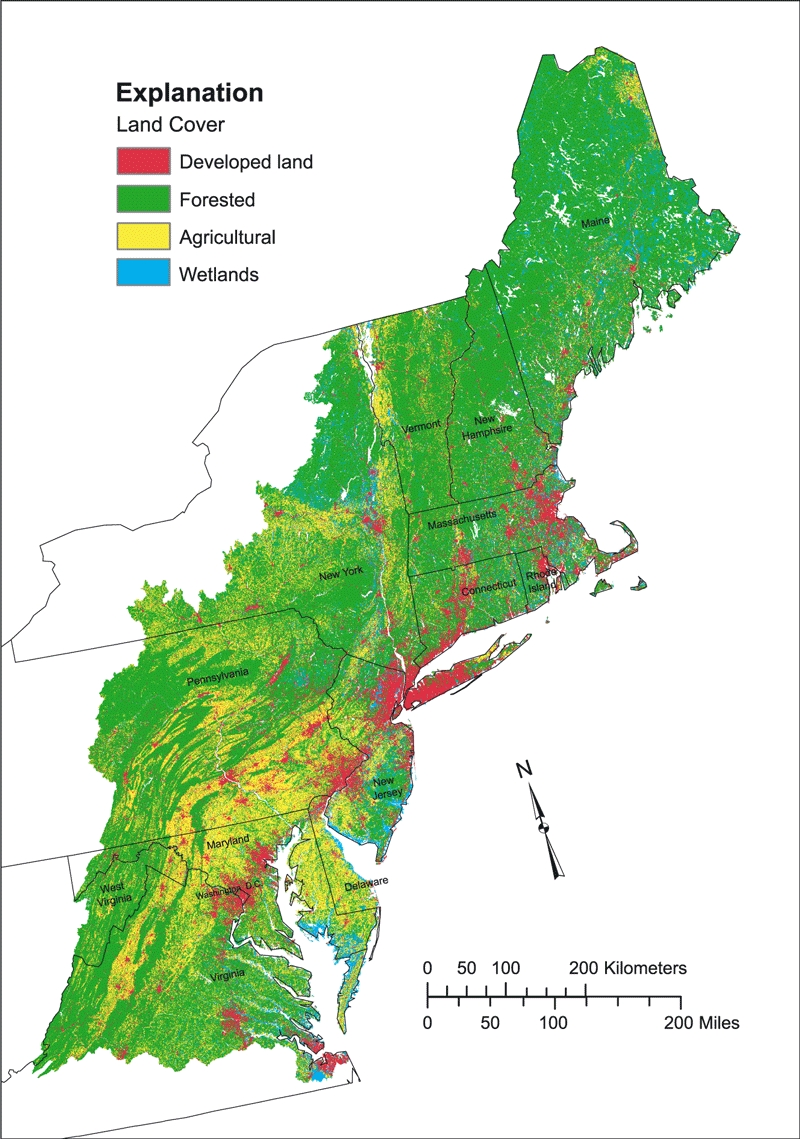
Major Land Cover Categories Within the Northeastern and Mid-Atlantic Regions Study Area (2001 National Land Cover Database).

Diffuse (nonpoint) nutrient sources are characterized in the SPARROW models through land-cover data and measures of agricultural activities, population density, and atmospheric deposition. Land cover was defined by the 2001 National Land Cover Database (NLCD) (Figure 2) that is available at a 30-m resolution grid (Fry *et al.*, 2009) and allocated to the catchment areas (Wieczorek and Lamotte, 2011). Major land-cover categories within the NE US include developed, forest, and agricultural land.

The atmospheric deposition dataset (Supplement B, Figure S2) used as input to the nitrogen SPARROW model is based on a simple inverse distance-weighted interpolation of the National Atmospheric Deposition Program/National Trend Network (NADP/NTN) data (USGS, 2000) following the technique used by Alexander *et al.* (2001). The resulting dataset includes estimates of the wet deposition of total inorganic nitrogen (TIN), nitrate (NO_3_^−^), and ammonium (NH_4_^+^) detrended to the 2002 base year (Wieczorek and Lamotte, 2011). The estimates are long-term averages that have been adjusted for long-term trends in order to represent 2002 conditions.

County- and state-based estimates of the nitrogen and phosphorus contents of fertilizers applied to agricultural lands and from livestock manure wastes also were tested and used as predictors. Source loadings for each catchment were determined from county-level application rates of fertilizer and manure that were spatially distributed based on agricultural land-cover NLCD categories (Wieczorek and Lamotte, 2011).

Land-to-water delivery variables tested in the model include soil permeability, land-surface slope, mean distance within the catchment to the stream, the percentage of streamflow coming from groundwater (Wolock, 2003), and mean annual climatic factors such as precipitation and temperature. Nutrient ecoregions and hydrologic landscape regions also were examined to determine if regional variations in land-to-water delivery of nutrients could be detected. The USEPA nutrient ecoregions (Omernik, 1995) are specifically aimed at factors that affect nutrients. The underlying concept is that a nutrient ecoregion contains within it similar soils, landforms, geology, climate, and ecological communities that process nutrients in similar ways. The SPARROW models are used to test the regional effect of these differences between ecoregions on the delivery of nutrients to the stream network. The inclusion of ecoregion terms is intended to improve the accuracy of the model predictions by accounting for subregional variability in flux that is not fully explained by the other explanatory variables.

For atmospheric deposition of nitrogen, a land-to-water delivery factor, specific to atmospheric deposition, was tested and used in the NE US nitrogen SPARROW model. This delivery factor is the ratio of nitrate to TIN within the wet atmospheric deposition. The rationale for the use of this ratio as a delivery factor is that the ammonium cations can be strongly adsorbed on soil particle or mineral surfaces and may be transported differently than nitrate anions, which are readily transported in water and stable over a considerable range of conditions (Hem, 1985). In addition, if the water from precipitation passes through an anaerobic environment before reaching the stream network, there is the potential for nitrate loss through denitrification. In the model, a higher ratio of nitrate to TIN, for example, is thus expected to be associated with a higher rate of delivery of atmospheric nitrogen to the streams.

Aquatic decay variables are tested in the models. In order to estimate instream nutrient losses, estimates of mean-annual flow and velocity are required. These data have been assigned to each flowline in the NHDPlus network (U.S. Environmental Agency and USGS, 2006, accessed at http://www.horizon-systems.com/nhdplus/). Instream decay rates are estimated on the basis of travel times within the stream segments and evaluated on the basis of statistical significance of the explained spatial variation in stream load.

For aquatic decay in lakes and reservoirs, the reciprocal hydraulic load (the ratio of the water surface area divided by the associated flow) is tested as the explanatory variable. Additional details on this formulation are given in Alexander *et al.* (2002) and Schwarz *et al.* (2006). The water in many lakes and reservoirs is compartmentalized because water enters via multiple tributaries, as depicted in the 1:100,000-scale NHD. SPARROW model estimates of nutrient loss can thus be determined for each lake or water body compartment, thus providing additional detail to model predictions and applications.

### Estimation Process

The estimation process involves calibrating SPARROW models by using established statistical procedures designed to optimize model fit by minimizing error between predicted and observed values of the monitored nutrient loads (Schwarz *et al.*, 2006). The calibrated models include only those explanatory variables that show statistically significant relations to the annual nutrient loads in streams. Bootstrap analysis was conducted to confirm estimation results. For the SPARROW models, the bootstrap analysis is based on the computation of 200 sets of model coefficients, which are estimated by resampling the data to generate 200 sets of data (Schwarz *et al.*, 2006). Source and aquatic decay variable coefficients were constrained to positive numbers in this process.

### Applications of the Models

The NE US nitrogen SPARROW model was used to estimate mean-annual nitrogen loadings and source allocations to estuaries within the NE US regions of the United States, specifically the nine largest rivers within the NE US. In order to preserve mass balance, a NLLS solution was used with no adjustments of predictions at monitoring sites. This information helps identify the relative contributions of different river basins to total nutrient loads, the sources of the loads within those basins, and where potential estuarine eutrophication may occur as a result of high nitrogen loads.

The NE US phosphorus SPARROW model was used to identify lakes and lake embayments that have already become eutrophic or are likely to become so as a result of phosphorus accumulation. A set of 13 large lakes and reservoirs was selected for analysis. These geographically distributed lakes all have water-surface areas greater than 18 km^2^, a watershed entirely within the United States, and represent a variety of land-cover and other physical characteristics. Lakes with land-use percentages similar to other selected lakes were excluded. Total annual delivered loads of phosphorus were then summed by lake or reservoir.

## Estimation Results

Estimation and bootstrap results for the NE US SPARROW models for nitrogen and phosphorus, including coefficient estimates, 90th percentile confidence intervals of the model coefficients, standard errors, and probability levels of significance, are presented in Table 1. Significant predictors for both models include (1) nutrient mass in permitted wastewater discharge; (2) the area of developed land; (3) nutrient mass in commercial fertilizer applied to agricultural land planted in corn, soybean, and alfalfa, plus, for nitrogen, estimates of nitrogen mass from nitrogen fixation by soybean and alfalfa; (4) nutrient mass in commercial fertilizer applied to agricultural land in other crops; and (5) nutrient mass in manure from livestock production. The nitrogen model has six source terms, five land-to-water delivery terms, and one instream attenuation term (Table 1), while the phosphorus model has six source terms, four land-to-water delivery terms, and one lake/reservoir loss term. The coefficient values have various physical interpretations. For a more detailed discussion of the nitrogen and phosphorus, model coefficients and their physical interpretation, see Supplements A and C of this journal article. A source specific to the nitrogen model, but not to the phosphorus model is wet atmospheric deposition of inorganic nitrogen (ammonium and nitrate). A source specific to the phosphorus model is the area of forested land.

**TABLE 1:** Estimation Results and Bootstrap Estimates for the Northeastern and Mid-Atlantic SPARROW Model for Total Nitrogen (TN) and Total Phosphorus (TP)

Nitrogen							

Nitrogen Parameters (units)	Coefficient Units	Model Coefficient (NLLS)	Lower 90% Confidence Interval for Coefficient	Upper 90% Confidence Interval for Coefficient	Standard Error of Coefficient	Probability Level (*p*-value)^b^	Nonparametric Bootstrap Estimate of Coefficient (mean)
Nitrogen sources^a^							
Developed land (km^2^)	kg/km^2^/year	1422	1062	1726	169	<0.001	1419
Wastewater discharge (kg/year)	Dimensionless	1.16	0.92	1.37	0.17	<0.001	1.16
Fertilizer applied to, and fixation from, agricultural land in corn / soybeans / alfalfa (kg/year)	Dimensionless	0.310	0.220	0.390	0.039	<0.001	0.314
Fertilizer applied to agricultural land in other crops (kg/year)	Dimensionless	0.186	−0.168	0.372	0.081	0.011	0.141
Manure from livestock production (kg/year)	Dimensionless	0.090	0.033	0.131	0.026	<0.001	0.087
Wet deposition of inorganic nitrogen (ammonia and nitrate), detrended (kg/year)	Dimensionless	0.279	0.218	0.338	0.028	<0.001	0.283
Land-to-water delivery							
Mean annual temperature (ln (°C))	Per ln (°C)	−0.864	−1.112	−0.637	0.118	<0.001	−0.877
Average overland flow distance to the stream channel (km)	km^−1^	−0.190	−0.247	−0.129	0.025	<0.001	−0.193
ln (ratio of nitrate to total inorganic nitrogen wet deposition)	Dimensionless	2.56	−0.09	4.95	1.21	0.035	2.42
Northern Piedmont Ecoregion indicator (0,1)	Dimensionless	0.422	0.289	0.576	0.073	<0.001	0.422
Valley and Ridge Ecoregion indicator (0,1)	Dimensionless	0.593	0.454	0.755	0.076	<0.001	0.602
Aquatic decay							
Time of travel in each stream reach where mean discharge <2.83 m^3^/s (days)	Per day	0.224	−0.031	0.447	0.144	0.060	0.216
MSE		0.12					
RMSE		0.35			*R*-squared load		0.97
Number of observations		363			*R*-squared yield		0.83

Phosphorus							

Phosphorus Parameters (units)	Coefficient Units	Model Coefficient (NLLS)	Lower 90% Confidence Interval for Coefficient	Upper 90% Confidence Interval for Coefficient	Standard Error of Coefficient	Probability Level (*p*-value)^*b*^	Nonparametric Bootstrap Estimate of Coefficient (mean)
Phosphorus sources^a^							
Developed land (km^2^)	kg/km^2^/year	106.3	69.1	132.4	14.2	<0.001	104.0
Forested land (km^2^)	kg/km^2^/year	11.4	7.7	14.9	1.7	<0.001	11.5
Wastewater discharge (kg/year)	Dimensionless	1.32	1.04	1.58	0.22	<0.001	1.31
Fertilizer applied to agricultural land in corn/soybeans/alfalfa (kg/year)	Dimensionless	0.070	0.035	0.104	0.019	<0.001	0.070
Fertilizer applied to agricultural land in other crops (kg/year)	Dimensionless	0.230	0.106	0.337	0.083	0.003	0.234
Manure from livestock production (kg/year)	Dimensionless	0.056	0.035	0.073	0.010	<0.001	0.055
Land-to-water delivery							
Percentage of streamflow coming from ground water (%)	Dimensionless	−0.996	−1.410	−0.620	0.236	<0.001	−1.035
Average overland flow distance to the stream channel (km)	(km^−1^)	−0.580	−0.763	−0.369	0.095	<0.001	−0.574
Northeastern Coastal Zone Ecoregion indicator (0,1)	Dimensionless	−0.543	−0.802	−0.290	0.178	0.003	−0.539
Eastern Great Lakes and Hudson Lowlands Ecoregion indicator (0,1)	Dimensionless	0.97	0.66	1.21	0.43	0.024	0.96
Aquatic decay							
Lake/reservoir loss; inverse hydraulic load (m/year)	(m/year)	2.69	−2.79	5.39	2.40	0.132	1.94
MSE		0.42					
RMSE		0.65			*R*-squared load		0.91
Number of observations		457			*R*-squared yield		0.60

Notes: NLLS, nonlinear-least-squares; MSE, mean square error; RMSE, root mean square error; *R*-squared, coefficient of determination; (0,1), equals 1 if within ecoregion.

a The source coefficients, which measure the mean rate of nutrient mass deliver to streams as a function of the source input units, are standardized to the mean of the land-to-water delivery variables. The sources with dimensionless coefficients multiplied by an exponential land-to-water delivery function quantify the proportion of available nutrient mass delivered to rivers.

b The reported *p*-values are one-sided values for the source and aquatic-decay variables and two-sided for the land-to-water delivery variables.

In general, the nutrient loads predicted by the model reasonably match the observed load as indicated by coefficients of determination (*R*^2^) of 0.97 and 0.91, and a root mean-squared error of 0.35 and 0.65, for the nitrogen and phosphorus models, respectively. The coefficient of determination is a measure of the fraction of variance in the load data (expressed in natural log units) that was accounted for by the independent variables used in the regression model. Therefore, the nitrogen model accounted for 97% of the variance in the log-transformed values of the mean annual load. Some of this variance, however, is the result of drainage area size and is not due to the relative intensity of nutrient generating activities. Large rivers tend to have larger loads of nitrogen than smaller rivers. “A high R-square, therefore, does not necessarily indicate the strength of the model within a smaller basin. Goodness of model fit for small basins might be better described by R-square of the logarithm of contaminant yield, R^2^-yield” (Schwarz *et al.*, 2006, pp. 97; equation 1.112). Adjusting for drainage-area scaling effects, the models explained 83 and 60% of the variance in nutrient yield for the nitrogen and phosphorus models, respectively (Table 1).

Bootstrap analysis results are presented in Table 1 to show the stability of the model coefficients. The close agreement between the NLLS and the bootstrap results provides confidence in the simpler NLLS models. Only the NLLS estimation models are used for further applications and analysis in this study.

**FIGURE 3:**
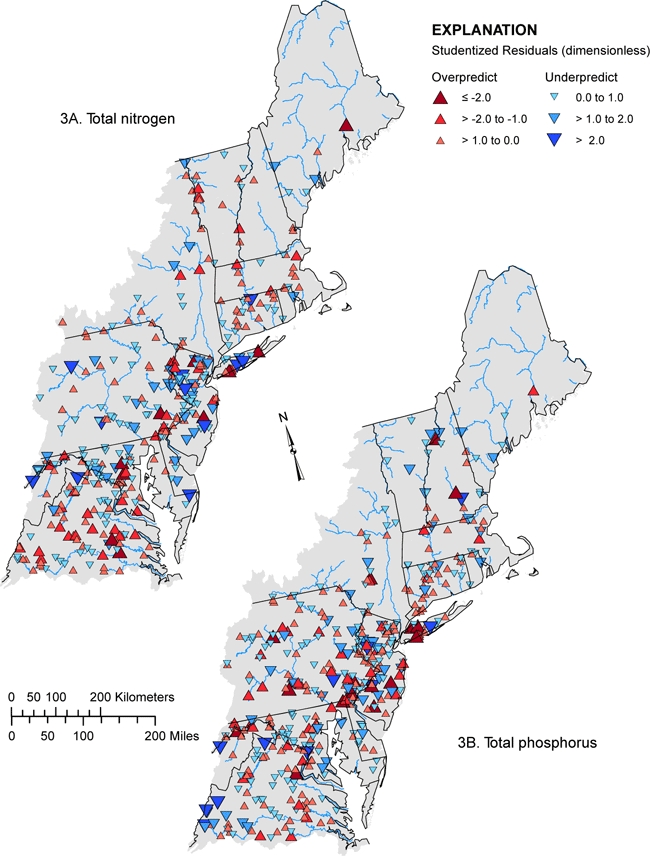
Model Residuals for Sites Used to Calibrate the SPARROW Models of (A) Total Nitrogen, and (B) Total Phosphorus. Studentized residuals are presented.

Residuals for the SPARROW models are presented in Figure 3. Positive residuals indicate areas in which the model under-predicts loads, and negative residuals areas in which the model over-predicts loads. For the nitrogen model residuals (Figure 3A), the model fits particularly well with no obvious regional-scale patterns, and the majority of the nitrogen residual magnitudes were between 1 and −1. For the phosphorus model residuals (Figure 3B), the model appears to fit well in that there are no obvious spatial patterns. However, compared to the nitrogen model, there are more residuals greater than 1 and less than −1. The greater range of phosphorus residuals (than nitrogen) is reflective of the smaller *R*^2^ of 0.91.

### Prediction Summary

**FIGURE 4:**
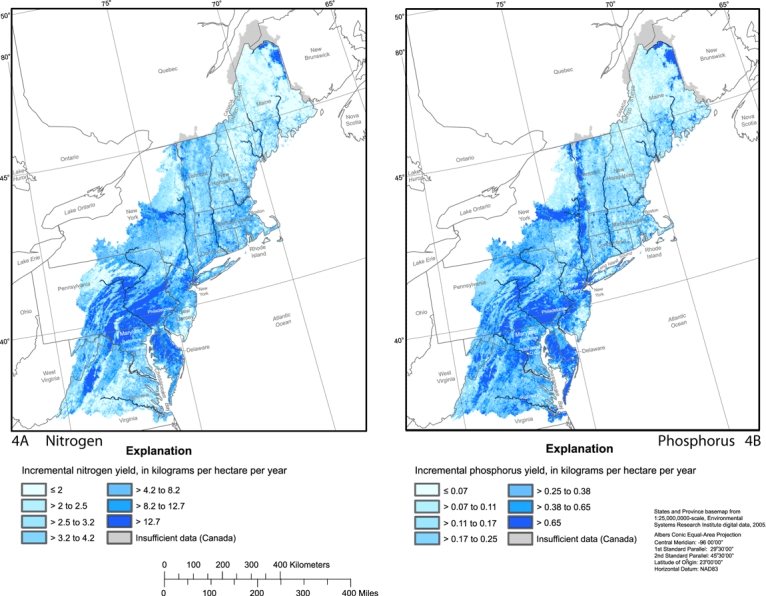
Nitrogen (A) and Phosphorus (B) Yields Predicted by the Northeastern and Mid-Atlantic Regions SPARROW Model. [Figure replacement made here after initial online publication, August 24, 2011]

Figure 4 presents a comparison of the predicted incremental yields for nitrogen (Figure 4A) and phosphorus (Figure 4B). Incremental yields are the yields associated with individual stream reaches (NHD flowlines) and their associated catchments. Areas of high nitrogen and phosphorus yield generally correlate to agricultural and developed lands (Figures 2 and 4). A summary of predicted yields and source shares by catchment is given in Table 2. The mean nitrogen and phosphorus yield, for all catchments, is 9.53 and 3.43 kg/ha/year, respectively. The amount of nitrogen or phosphorus generated within a given incremental catchment that is ultimately delivered to the Atlantic Ocean (or Canada) is 5 and 6% less than this respectively. This finding indicates that in the NE US, total attenuation losses within the stream, lakes and reservoirs are small. This is in contrast to much larger attenuation rates that must occur prior to reaching the stream network. The model does not explicitly quantify terrestrial losses, however, the coefficient of 0.28 on the atmospheric deposition variable (Table 1) is indicative of a 72% loss prior to ever reaching the streams.

**TABLE 2:** Summary Statistics of Yields and Source Shares From 193,336 NHDPlus Catchments Within the Northeastern and Mid-Atlantic Regions of the United States

	Total Nitrogen (TN)							Total Phosphorus (TP)						
		
Variable	Mean	SD	10th Percentile	25th Percentile	Med	75th Percentile	90th Percentile	Mean	SD	10th Percentile	25th Percentile	Med	75th Percentile	90th Percentile
Yield^1^ (kg/ha/year)														
	9.5	273.5	1.4	2.1	3.7	7.7	13.2	3.43	38.33	0.06	0.13	0.25	0.54	1.02
Percentile														
Atmospheric deposition	49.9	34.0	11.1	18.7	40.5	86.5	100.0	NA	NA	NA	NA	NA	NA	NA
Manure	6.0	8.9	0.0	0.0	1.8	8.9	18.3	14.4	20.0	0.0	0.0	4.2	22.4	46.5
Corn, Soy, Alfalfa	18.6	24.3	0.0	0.0	5.4	33.6	57.8	9.8	17.4	0.0	0.0	1.6	11.7	29.5
Farm fertilizer other crops	3.9	7.7	0.0	0.0	0.7	5.1	10.2	7.3	13.4	0.0	0.0	1.0	9.6	21.0
Point sources	0.3	5.3	0.0	0.0	0.0	0.0	0.0	0.4	5.7	0.0	0.0	0.0	0.0	0.0
Developed land	21.3	25.8	0.0	0.0	10.7	33.4	65.9	24.9	30.1	0.0	0.0	12.6	38.7	79.0
Forested Land	NA	NA	NA	NA	NA	NA	NA	43.2	37.8	1.5	7.9	31.1	83.7	100.0

Notes: Med, median (50th percentile); SD, standard deviation; NA, not applicable.

Incremental yields represent the load generated within an incremental watershed (the area that drains directly to a stream reach without passing through another stream reach) divided by the area of the incremental watershed.

Source shares represent the contribution from each source as a percentage of the incremental yield.

1 The amount of TN or TP generated within a given incremental catchment that is delivered to the catchment outlet.

The mean agricultural source shares (sum of manure and fertilizer sources), by catchment, are similar for nitrogen and phosphorus models, both contributing, on average, about 30% of the respective nutrient. Source shares, by catchment, are the percentages of each nutrient attributed to each source of the total nutrient load that reaches the stream from within that reach's catchment. The corn, soybean, and alfalfa crop group, however, accounts for more of the source share for nitrogen (18.6%) than for phosphorus (9.8%). Mean source shares by catchment from point sources and developed lands are about the same for both nutrient models.

## Application Results

### Nitrogen Model – Estuarine Application

The NE US nitrogen SPARROW model was used to predict nitrogen loads and source allocations delivered to the estuaries of the nine largest rivers within the NE US (Figure 1) (Table 3). Agricultural sources contribute the largest percentage (37%) of the total combined load of nitrogen delivered to the estuaries of the nine large estuary systems analyzed. Agricultural sources dominate delivered loads from the Susquehanna (59%) and the Potomac (45%) watersheds. Because the Susquehanna watershed is by far the largest watershed within the NE US, it has the greatest influence on the overall source share percentages for the NE US. Agricultural sources of nitrogen also are high in the watersheds of the Hudson (28%), Delaware (29%), and James (20%) rivers. Model simulation results indicate that the crop group of corn, soybean, and alfalfa (grown in rotation) is an especially important source of nitrogen to these estuaries (Table 3). This is particularly so for the Susquehanna, where this crop group accounts for 39% of the nitrogen load reaching the Chesapeake Bay estuary.

**TABLE 3:** Predicted Nitrogen Loads by Drainage Basin Delivered to its Estuary Within Northeastern and Mid-Atlantic SPARROW Model Area

		Predicted Percent of Nitrogen Load From Various Sources								
		
			Total Nitrogen (metric tons per year)^1^					Agricultural		
								
River Basin	State	Drainage Area (km^2^)		Atmospheric Deposition	Point Sources	Developed Lands	Sum of Agriculture Sources	Corn Soy Alfalfa	Other Fertilizer	Manure
James		26,779	15,864	15.7	50.1	14.1	20.0	11.4	1.2	7.4
	Virginia		15,809	15.6	50.3	14.1	20.0	11.4	1.2	7.4
	West Virginia		55	44.8	0.0	20.6	34.5	19.1	1.9	13.5
Potomac		37,965	40,569	12.6	29.6	13.1	44.7	26.8	2.7	15.2
	Virginia		16,995	9.0	35.4	13.6	42.0	21.9	1.7	18.4
	Maryland		8,881	13.3	13.3	16.7	56.7	45.4	3.0	8.3
	West Virginia		5,335	32.3	2.4	16.4	48.9	20.9	2.7	25.3
	District of Columbia		4,651	0.3	97.5	2.2	0.0	0.0	0.0	0.0
	Pennsylvania		4,708	14.6	2.8	11.4	71.2	42.7	8.2	20.3
Susquehanna		71,200	66,280	19.0	8.7	12.9	59.4	39.5	5.4	14.5
	Pennsylvania		54,961	18.0	8.4	13.3	60.3	39.9	5.2	15.1
	New York		10,464	24.8	11.0	11.8	52.5	34.2	6.7	11.6
	Maryland		855	12.3	0.0	3.5	84.2	75.3	0.8	8.2
Delaware		30,612	45,849	11.8	45.8	13.7	28.7	21.5	3.8	3.5
	Pennsylvania		33,150	10.1	46.4	13.8	29.7	22.4	3.5	3.8
	New Jersey		9,441	9.8	53.2	12.0	24.9	17.7	5.6	1.7
	New York		1,764	55.2	5.6	15.6	23.6	13.2	2.8	7.7
	Delaware		1,464	8.5	35.3	19.8	36.4	35.2	0.4	0.8
	Maryland		30	8.0	0.0	11.9	80.0	71.9	3.2	4.9
Hudson		34,612	26,054	23.3	34.8	13.8	28.0	19.8	3.6	4.7
	New York		22,442	25.3	28.7	14.8	31.3	22.1	4.0	5.2
	New Jersey		2,904	4.2	85.9	4.7	5.2	3.8	0.5	0.9
	Vermont		399	48.4	11.2	20.0	20.3	13.4	2.3	4.7
	Massachusetts		296	30.6	31.9	21.1	16.3	10.1	2.2	4.0
	Connecticut		13	38.1	0.0	55.6	6.2	1.2	3.8	1.3
Connecticut		29,166	15,641	33.5	26.9	22.6	17.0	7.5	5.2	4.3
	Massachusetts		4,553	23.9	33.2	22.6	20.3	9.7	7.9	2.7
	Connecticut		4,431	11.0	54.7	21.8	12.5	6.1	5.2	1.2
	Vermont		3,795	54.0	2.5	22.8	20.7	7.7	3.9	9.1
	New Hampshire		2,790	55.8	6.1	24.1	14.1	6.3	2.6	5.2
	Quebec		73	94.6	0.0	3.0	2.4	1.0	0.5	0.9
	Maine		0	88.6	0.0	11.2	0.2	0.0	0.0	0.2
Long Island Sound TMDL^2^ study – Connecticut River (to compare with SPARROW)			16,167	32.7	30.2	NA	NA	NA	NA	NA
	Connecticut		4,788	15.8	53.1	NA	NA	NA	NA	NA
Merrimack		12,950	8,229	24.9	37.8	29.5	7.8	3.9	2.3	1.6
	New Hampshire		4,383	38.4	23.6	30.0	7.9	4.0	1.8	2.1
	Massachusetts		3,846	9.3	54.3	28.8	7.7	3.7	2.9	1.0
Kennebec		24,770	6,841	53.7	11.4	23.0	11.9	4.0	3.6	4.3
	Maine		6,370	52.1	11.8	23.4	12.7	4.3	3.9	4.6
	New Hampshire		471	74.4	6.6	17.4	1.6	0.6	0.3	0.7
Penobscot		21,908	4,912	66.4	4.3	17.7	11.6	3.3	6.2	2.1
	Maine		4,912	66.4	4.3	17.7	11.6	3.3	6.2	2.1

Notes: NA, not applicable; TMDL, total maximum daily load.

1 Predicted loads are based solely on the estimated SPARROW model and are not adjusted at monitored reaches to equal the monitored load.

2 1988-1990 time period; New York State Department of Environmental Conservation and Connecticut Department of Environmental Protection, 2000.

Point sources account for 28% of the total nitrogen load delivered to the nine large river estuaries combined. Of these large rivers, six have point sources whose contributions to the total load of nitrogen delivered to their estuaries equal or exceed 25%. These include the James (50%), the Delaware (46%), the Hudson (35%), the Merrimack (38%), the Potomac (30%), and the Connecticut (27%) rivers (Table 3).

Atmospheric deposition contributes 20% of the total combined load of nitrogen delivered to the nine large river estuaries listed in Table 3. The atmospheric sources modeled represent regional sources (not local urban) and may not fully account for dry deposition contributions. Supplement B provides additional information on the atmospheric sources. Also, developed land likely includes contributions of nitrogen deposition from local vehicle emissions. The atmospheric deposition of nitrogen delivered to the nine large river estuaries ranged from as little as 12-19% of the total delivered loads in the southern watersheds (those of the Potomac, James, Delaware, and Susquehanna rivers) to as high as 54-66% in the extreme northeastern U.S. watersheds (those of the Kennebec and Penobscot rivers). The atmospheric contributions of nitrogen to the Hudson, Connecticut, and Merrimack rivers are intermediate, at 23, 34, and 25%, respectively, of the total delivered load. Although atmospheric deposition of nitrogen is generally highest in the central portion of the NE US and decreases northward, it is the largest source of nitrogen delivered to the estuaries for the watersheds of the Connecticut, the Kennebec, and the Penobscot rivers, where loads from human-related sources, such as agricultural practices, tend to be smaller than in the more southerly watersheds.

**FIGURE 5:**
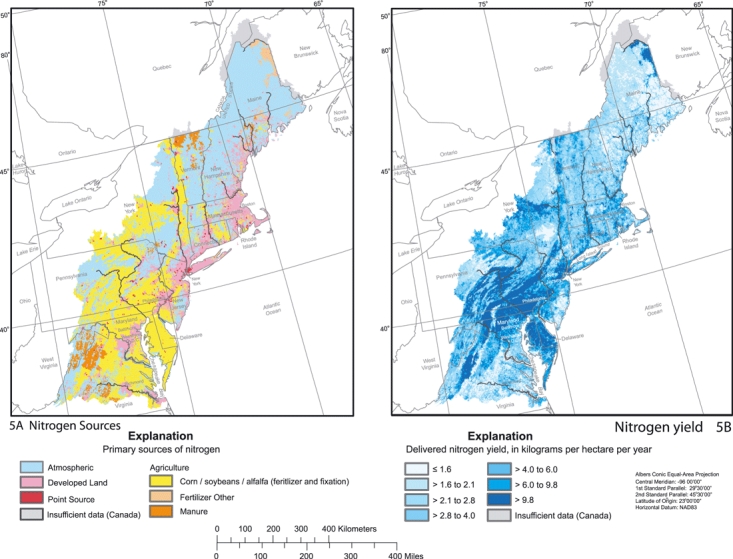
Primary Sources of Nitrogen (A), and Nitrogen Yields, Delivered to Estuaries (or end of modeled area) (B) Predicted by the Northeastern and Mid-Atlantic Regions Nitrogen SPARROW Model.

An overview of the role of primary sources of nitrogen and the yields of nitrogen delivered to estuaries (or the model boundary) is shown in Figure 5. The primary or leading source of nitrogen within each catchment, predicted by the NE US SPARROW nitrogen model, is shown in Figure 5A, and the yields of nitrogen delivered to estuaries (or boundary of modeled area) from each of the catchments is shown in Figure 5B. Figure 5A indicates that agricultural and developed lands are the major source of nitrogen in water in much of the NE US. However, atmospheric deposition is also an important source of nitrogen in large parts of the NE US, especially in mountainous areas and in the northern half of the region. Also the nitrogen yields are greatest in those parts of the NE US in which the crop group corn, soybean, and alfalfa is grown and with developed lands.

#### Comparison With Other Studies of Predicted Nitrogen Loads to Estuaries

The usefulness of SPARROW model applications to estimates of TMDL is demonstrated by comparing the NE US nitrogen SPARROW simulation results to data compiled by New York State Department of Environmental Conservation and Connecticut Department of Environmental Protection (2000) for the Long Island Sound TMDL (Tables 3 and 4, respectively). As part of the Long Island Sound TMDL study, total nitrogen load delivered to Long Island Sound, from the Connecticut River, was estimated for the 1988-1990 time period. These estimates serve as a base level from which improvements could be measured. The TMDL analysis also included estimates of nitrogen loadings from the Connecticut portion of the watershed, as well as estimates from all upstream sources. Additionally the loads were apportioned to various sources, including atmospheric deposition and point sources. (Other sources listed in the TMDL report are more general categories “terrestrial” or background sources “pre colonial”). The NE US SPARROW results compare favorably with those of the TMDL study (Tables 3 and 4). This is true for the entire watershed, as well as for source shares within the Connecticut River watershed and source shares within the State of Connecticut.

**TABLE 4:** Predicted Annual Total Nitrogen (TN) Loads for Nine Coastal Rivers From the Northeastern and Mid-Atlantic Regions SPARROW Model Compared With Similar Estimates From Previously Published SPARROW Models and the NOAA National Estuarine Eutrophication Assessment (NEEA). Load estimates are for various time periods as noted

	TN Load (metric tons per year)											
	
	Northeastern and Mid-Atlantic Regions SPARROW^1^,^2^		Long Island Sound TMDL Study^3^		New England SPARROW^2^,^4^		Chesapeake Bay SPARROW^2^,^5^		National SPARROW^2^,^6^		NEEA^7^	
						
River	TN (metric tons per year)^1^	Drainage Area (km^2^)	TN (metric tons per year)^1^	Drainage Area (km^2^)	TN (metric tons per year)^1^	Drainage Area (km^2^)	TN (metric tons per year)^1^	Drainage Area (km^2^)	TN (metric tons per year)^1^	Drainage Area (km^2^)	TN (metric tons per year)^1^	Drainage Area (km^2^)
Penobscot	4,912	21,908	NA		3,625	21,908	NA		8,643	23,149	9,740)^8^	23,241
Kennebec	6,841	24,770	NA		7,377	24,770	NA		11,277	24,614	12,400)^8^	24,601
Merrimack	8,229	12,950	NA		9,425	12,930	NA		9,098	12,641	9,940	13,002
Connecticut	15,641	29,166	16,167	29,171	15,951	29,166	NA		21,843	29,067	15,560	28,891
Hudson	26,054	34,612	NA		NA		NA		70,232)^9^	41,793	71,900)^9^	41,603
Delaware	45,849	30,612	NA		NA		NA		46,971	33,777	45,540	33,254
Susquehanna	66,280	71,200	NA		NA		38,943	71,124	57,120	70,189	NA	
Potomac	40,569	37,965	NA		NA		34,083	36,980	38,156	37,392	33,770	36,804
James	15,864	26,779	NA		NA		18,238	26,193	17,050	26,882	13,370)^10^	26,101

Note: TMDL, total maximum daily load; NA, not applicable.

1 2002 base year; this study (Table 3).

2 Predicted loads are based solely on the SPARROW nonlinear-least-squares simulations. Monitored loads are not substituted for the predicted loads at monitored reaches.

3 1988-1990. New York State Department of Environmental Conservation and Connecticut Department of Environmental Protection, 2000.

4 1992-1993 period; predicted loads, computed using a nonlinear-least-squares simulation, are within 2% of the predictions from the parametric-bootstrap simulation, published in Moore *et al.*, 2004.

5 1997; John Brakebill, USGS, written communication, 2011; Brakebill and Preston, 2004.

6 2002; Alexander *et al.*, 2008.

7 From 1994-2004 NEEA; Bricker *et al.*, 2007.

8 From 1982-1991 NEEA; Bricker *et al.*, 1999.

9 The national SPARROW and NEEA load predictions for the Hudson River include the Raritan Bay (including point source discharges to the bay and additional drainage including the Raritan, Rahway, Passaic, and Hackensack rivers) while the Northeastern and Mid-Atlantic Regions SPARROW (NHD terminal flowline for the Hudson River) does not include this additional drainage.

10 Dissolved inorganic nitrogen only.

Simulations made with three previously published SPARROW models (Table 4), and data from the National Estuarine Eutrophication Assessment (NEEA), also provide estimates of nitrogen loads for some or all of the nine coastal rivers listed in Table 4. These earlier estimates are provided for additional comparison, recognizing that the time periods and methods differ among the estimates (see Table 4 notes). Drainage area also differs and accounts for some of the differences in loads. Table 4 provides drainage areas associated with each study and river. For the NE US SPARROW, this study, the drainage areas and loads are associated with the terminal reach of the NHD (1:100,000 scale). Simulations with drainage areas larger, or smaller, than the NE US SPARROW are indicative that the loads have been accumulated either further out into the estuary or not as far out into the estuary, respectively.

The first model used for comparison is a SPARROW model of the New England region (Moore *et al.*, 2004) for the period 1992-1993, a decade earlier than the NE US model. The time frame for this earlier SPARROW model matches that for the Connecticut River TMDL analysis for Long Island Sound more closely than does the NE US SPARROW model and the results also compare more closely than those for the NE US SPARROW (Table 4).

Two additional SPARROW models, used for comparison (Table 4), are a Chesapeake Bay drainage SPARROW and a national SPARROW model. Brakebill and Preston (2004) constructed a SPARROW model of the Chesapeake Bay drainage for a 1997 base year. Three national SPARROW models have been produced to represent different time frames (Smith *et al.*, 1997; Alexander *et al.*, 2001, 2008). The most recent national estimates (Alexander *et al.*, 2008) are for 2002 conditions and are the ones used for comparison (Table 4). One noteworthy methodological difference between the NE US and New England SPARROW models compared to the Chesapeake Bay and national-scale SPARROW models is that the former utilize the more spatially detailed NHD stream network (see above and Brakebill *et al.*, this issue). The number of monitoring sites increases if the NHD stream network is used, because many monitoring sites are on smaller streams that are included in the NE US model. The last column in Table 4 presents nitrogen load estimates for the nine coastal rivers as reported in the NEEA, conducted for the period 1994-2004, where available, or 1982-1991, otherwise. These surveys, reported in the NEEA, are conducted by the National Oceanic and Atmospheric Administration and make use of monitoring data and information from a variety of local sources.

There are no clear patterns in the differences in load estimates for the six studies (Table 4) except that the estimates from New England and NE US SPARROW model studies are in close agreement with those of the Connecticut River – Long Island Sound TMDL study. Also, on average, the NE US model estimates are in closer agreement with those from the New England model than with those from the other models, despite the fact that the latter New England model represents a decade earlier time period. The larger disagreement between the NE US model estimates and those from the national-scale model may well be explained by the lower accuracy of the latter (mean square error (MSE) equals 0.3054). Drainage area also results in a large difference, in predicted loads for the Hudson River in that the national SPARROW load predictions include more high population drainage in and around New York City and nearby parts of New Jersey. Disagreement between the NE US model estimates and those from the Chesapeake Bay SPARROW may be the consequence of differences in the reporting period, the resolution of the stream network, the geographic extent of the models, and differences in model input data, including both the dependent (nitrogen stream loads at monitoring stations) and explanatory data, such as point sources and agricultural sources. The larger disagreement between the NE US model estimates and those from the NEEA surveys are likely the consequence of differences in both reporting period and methodology of the studies. For the Hudson River, the NEEA surveyed drainage area is much larger than that of the NE US SPARROW model (Table 4), but similar to the national SPARROW model. This is again a major cause of the difference in predicted loads (see Table 4 notes).

### Phosphorus Model – Lacustrine Application

The NE US SPARROW phosphorus model was used to predict phosphorus loads and source allocation for 13 large lakes/reservoirs within the NE US (Table 5). The 13 selected lakes/reservoirs can be grouped into two major categories: (1) those along the Susquehanna River with drainage areas greater than 67,000 km^2^; and (2) those with drainage areas smaller than 5,000 km^2^.

**TABLE 5:** Predicted Phosphorus Loads by Lake Watershed Delivered to Selected Lakes Within Northeastern and Mid-Atlantic SPARROW Model Area

								Predicted Percent of Phosphorus Load From Various Sources						
								
Lake/Reservoir Basin	State or States	Lake-Surface Area (km^2^)	Drainage Area (km^2^)	Total Phosphorus Entering the Lake (metric tons per year)^1^	Phosphorus Loss (accumulation) Within the Lake (metric tons per year)^1^	Percent Loss (accumulation) Within the Lake	Predicted Flow-Weighted Concentration Leaving the Lake (mg/l)	Point Sources	Forested Land	Developed Lands	Total Agriculture	Fertilizer Applied to Corn or Soy	Other Fertilizer	Manure
Lake Anna	Virginia	53	884	27	9	31	0.060	0.0	22.1	8.0	69.9	20.5	2.0	47.4
Conowingo Reservoir	Pennsylvania, New York, Maryland	16	70,131	3,741	12	0.3	0.130	18.6	14.0	13.9	53.4	11.5	9.2	32.7
Lake Aldred	Pennsylvania, New York	10	69,346	3,679	5	0.1	0.130	18.9	14.2	14.2	52.7	11.2	9.2	32.2
Lake Clarke	Pennsylvania, New York	30	67,590	3,346	18	0.5	0.121	19.0	15.6	14.9	50.5	10.8	9.8	29.9
Cannonsville Reservoir	New York	19	1,177	28	2	6.2	0.043	7.3	31.7	15.5	45.5	6.1	7.8	31.6
Great Sacandaga Lake	New York	101	2,701	37	6	15.7	0.022	0.0	68.0	22.7	9.3	1.4	2.3	5.5
Lake George	New York	115	604	6	4	55.2	0.010	9.8	56.0	29.7	4.5	0.9	0.8	2.8
Quabbin Reservoir	Massachusetts	95	485	5	3	59.5	0.002	0.0	56.3	28.2	15.5	2.7	7.6	5.1
Lake Winnipesaukee	New Hampshire	183	962	9	4	46.3	0.009	0.0	39.0	55.7	5.4	0.6	1.2	3.6
Sebago Lake	Maine	123	1,105	12	3	24.4	0.014	0.0	47.6	37.0	15.4	1.5	10.1	3.8
Moosehead Lake	Maine	302	3,292	19	4	20.1	0.010	0.0	89.1	10.0	0.9	0.0	0.0	0.9
Twin – Pemadumcook Lake	Maine	75	4,929	26	2	5.8	0.012	0.0	94.7	4.8	0.5	0.0	0.1	0.4
Long Lake	Maine	46	1,254	7	1	13.0	0.011	0.0	83.8	8.2	8.0	0.1	7.7	0.2

1 Predicted loads are based solely on the estimated SPARROW model and are not adjusted at monitored reaches to equal the monitored load.

**FIGURE 6:**
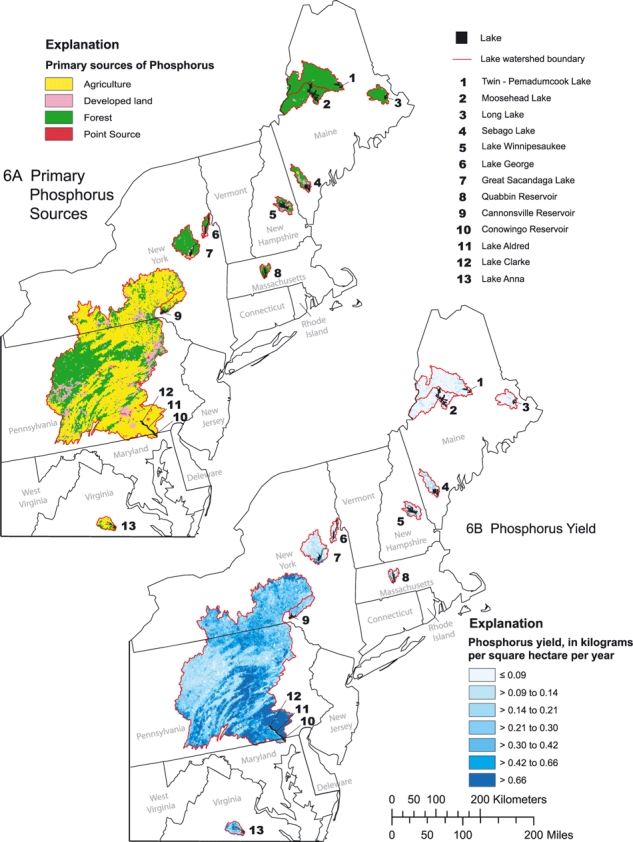
Primary Sources of Phosphorus (A), and Phosphorus Yields, Delivered to Selected Lakes (B) Predicted by the Northeastern and Mid-Atlantic Regions Nitrogen SPARROW Model.

The first category, those with large drainage areas, consist of a series of three long, narrow reservoirs that extend for 51 km end to end, along the southern, downstream end of the Susquehanna River – Lake Clarke at the upstream end, Lake Aldred in the middle, and Conowingo Reservoir at the downstream end (Figure 6) (Table 5). The dam for Conowingo Reservoir is about 16 km upstream of where the Susquehanna River enters Chesapeake Bay. The phosphorus loads and concentrations are higher for these three lakes/reservoirs than for the other 10 examined, all of which are in smaller watersheds. The percentage of the total phosphorus delivered to each lake that is contributed by agricultural sources is similar among the three lakes. There are, however, pronounced differences between the percentage of nitrogen and phosphorus contributed to the lakes/reservoirs by the various types of agriculture source (animal *vs.* crops). Agriculture contributes between 50 and 53% of the total phosphorus load to each lake (Table 5), with phosphorus mass in manure from livestock production identified as contributing the highest percentage, from 30 to 33% of the total phosphorus load for each lake. For nitrogen, the primary source identified for this area was the crop group of corn, soybean, and alfalfa (fertilizer plus fixation) (Figure 5). Model allocations indicate that only 14-16% of these phosphorus loads delivered to the lakes/reservoirs are from forested lands, while developed lands produce about 14-15% of the delivered load. This finding contrasts with predictions for many of the lakes/reservoirs with smaller watersheds, where most of the phosphorus is attributed to forested lands. Also in stark contrast to the lakes/reservoirs with smaller watersheds, the model predicts that nearly all of the phosphorus that enters these three lakes also leaves the lakes – that is, there is little potential for future long-term retention of phosphorus in the lakes beyond what may already be in storage. Less than 1% of the phosphorus that enters these lakes is predicted to remain in the lakes. For the upper two lakes, this is consistent with investigations of nutrient and sediment storage that conclude that Lakes Clarke and Aldred have virtually reached their nutrient storage capacity. The lower Conowingo Reservoir, created by a dam built in 1928, is also rapidly approaching its sediment and nutrient storage capacity (Langland, 1998). Currently, however, Conowingo Reservoir is still trapping a large percentage of the phosphorus entering the lake (Langland, 1998), and until its capacity is reached the reservoir is not responding as the regionally calibrated NE US SPARROW model would predict. This discrepancy is caused by the fact that the model estimates a loss coefficient that describes the net loss regionally and is not designed to be sensitive to any single reservoir. There is the potential to improve the models by the incorporation of predictor variables related to a wide variety of reservoir characteristics such as age, capacity, and management practices.

The second category of lakes/reservoirs comprises those with watersheds less than 5,000 km^2^. All 10 lakes/reservoirs in this category have model-predicted concentrations of phosphorus that are considerably lower than those of the lakes/reservoirs at the downstream end of the Susquehanna River.

#### Comparison of SPARROW Predictions to Measured Concentrations of Phosphorus

The relation between SPARROW model predictions and measured phosphorus concentrations in lakes/reservoirs was explored as an application of the NE US SPARROW phosphorus model. For this comparison, a subset of the National Lake Assessment (NLA) data was made available (Henry Walker, USEPA, written communication, 2009). In the NLA standardized protocols were used to collect physical, biological, and chemical data at more than 1,000 lakes in the continental United States during the summer of 2007. In this regard the values (instantaneous summer measurements) are not directly equivalent to SPARROW estimates (mean-annual estimates). Lakes more than 4 ha in area and greater than 1 m in depth were randomly selected from the NHD following a spatially balanced, probabilistic design that was stratified by lake size, ecoregion, and state. For each stratum, lakes were assigned a sampling weight (the inverse of the probability that any given lake will be chosen for sampling) that is used to make unbiased estimates of lake conditions at the state, region, and national levels (http://www.epa.gov/lakessurvey/; Stevens and Olsen, 2004; Olsen *et al.*, 2009).

**FIGURE 7:**
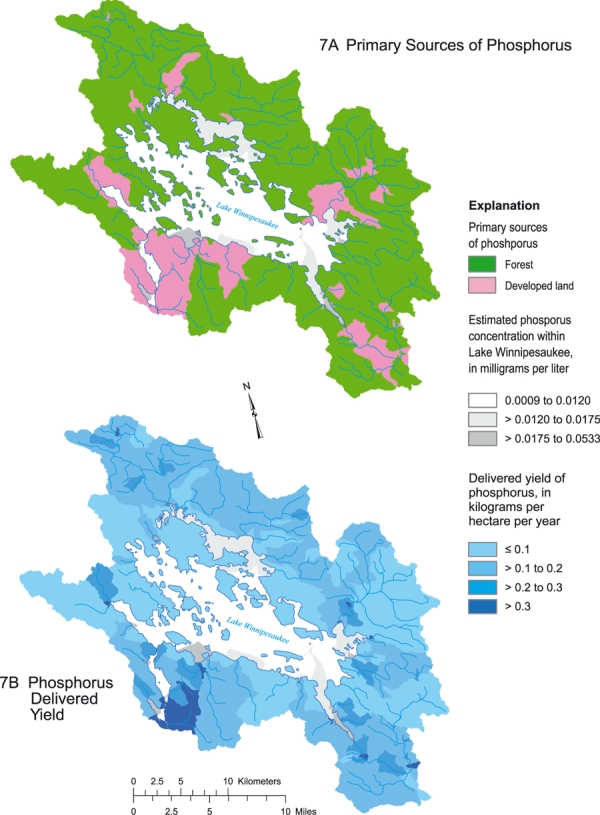
Primary Sources of Phosphorus (A), Lake Concentrations, and Phosphorus Yields (B) Delivered to Lake Winnipesaukee, New Hampshire, Predicted by the Northeastern and Mid-Atlantic Regions Phosphorus SPARROW Model.

The SPARROW model is calibrated to stream and river conditions, and thus was developed separate from lake concentration data. However, SPARROW models can indirectly predict concentrations within the lakes/reservoirs since the stream concentrations entering and leaving the lakes are estimated. These lake concentrations, independently predicted by SPARROW, can be compared with actual measured lake concentrations. Even within lakes, the SPARROW mean annual flow-weighted concentrations can be estimated by dividing the predicted annual load by the estimated annual flow for that reach. Annual flow data from NHDPlus were used in this calculation to predict concentration. In addition, SPARROW attenuates the predicted loads entering the lake in this process by estimating a coefficient that provides the best fit to the entire collection of reservoirs across the whole region, and then using the coefficient to estimate the nutrient loss or accumulation within each lake/reservoir. This is done by using the variables: (1) water surface area, and (2) the mean annual flow of water through the lake/reservoir, in order to compute the hydraulic load. SPARROW model predictions of concentration (mean annual flow-weighted concentration) are computed for each “artificial path” within all lakes/reservoirs in the network. The artificial path associated with the 2007 NLA sample location is then used to compare the concentrations SPARROW predicted to the independently observed data. (Artificial paths are transport reaches that facilitate the routing of flow and nutrients in the model through lakes/reservoirs). Figure 7 provides an example of how some lakes are subdivided by the NHDPlus catchments as well as the resulting SPARROW predictions.

**FIGURE 8:**
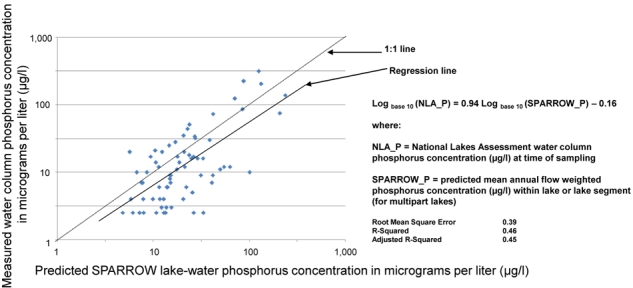
Comparison of Independently Predicted SPARROW Lake-Water Phosphorus Concentration to Measured Phosphorus Concentrations From the National Lake Assessment (U.S. Environmental Protection Agency, 2009).

SPARROW-predicted concentrations of phosphorus were compared to corresponding measured 2007 NLA data as an independent verification of model results. A least squares regression analysis of log-transformed SPARROW-predicted phosphorus concentration and log-transformed 2007 NLA data (Figure 8) indicated a linear relation between the two datasets (*R*^2^ = 0.46). The log transformation was made because residuals are log normally distributed, and base 10 was used to make the units more interpretable. For this analysis, values below the detection limit of 2.5 μg/l were set equal to the detection limit.

A least squares regression analysis yielded the results shown in Figure 8. Log_10_ was used to convert both the measured water column concentrations (at the time of sampling) and the independent SPARROW-predicted lake-water phosphorus concentration. In developing this statistical relation, it is possible to use, for comparison, the observed concentrations from either the SPARROW-predicted concentration at the lake outlet or at the segmented portion of the lake. The relation presented in Figure 8 is based on SPARROW predictions using the segmented approach. Observed water column concentrations are compared to the SPARROW predictions for the appropriate lake segment.

For lakes, the independently predicted SPARROW model phosphorus concentrations relate well to observed conditions, explaining 46% of the variance in observed data with approximately a one-to-one relation. The root MSE of 0.39 in log-space does indicate that the relation has a great deal of scatter in the residuals. However, the results do indicate that SPARROW could be used as a tool that allows for strategic monitoring of lakes likely to have high concentrations. This statistical comparison works reasonably well because the comparison encompasses a wide range of lake concentrations and lake conditions. The relation is only valid at the regional scale and would not be applicable to local scale models. A comparison of the data or the regression line to a 1:1 line (Figure 8) shows that the mean-annual flow-weighted SPARROW predictions are generally higher than the instantaneous summertime measurements, perhaps reflecting phosphorus uptake by aquatic biota during the summer season.

**FIGURE 9:**
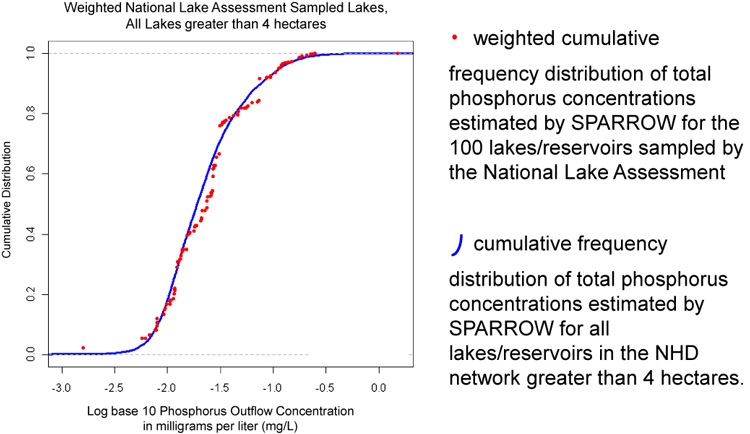
Comparison of Cumulative Distributions of SPARROW Predicted Phosphorus Concentrations for Weighted National Lake Assessment Sampled Lakes (U.S. Environmental Protection Agency, 2009) *vs.* All Lakes Within the Northeastern and Mid-Atlantic Regions of the United States Greater Than 4 ha Surface Area.

We also examine the data to determine if the NLA sampled lakes are representative of the full population of lakes. Figure 9 includes the weighted cumulative frequency distribution of phosphorus concentrations estimated by SPARROW for the 100 lakes/reservoirs sampled by the NLA. Also included is the cumulative frequency distribution for the estimated flow weighted annual phosphorus concentration at the lake outlet for all lakes/reservoirs in the NHD network that are greater than 4 ha. Visually, the two frequency distributions are nearly indistinguishable, and a statistical analysis, the Wilcoxon rank-sum nonparametric test (Helsel and Hirsch, 1992; SAS Institute, Inc., 1999) confirmed that they were statistically indistinguishable (not weighted, *p* = 0.21; weighted, *p* = 0.71). The SPARROW model results indicate that the probabilistic sampling design of the 2007 NLA data is representative of the full population of lakes in the Northeast given the desired stratification.

## Discussion

The NE US SPARROW models for nitrogen and for phosphorus have similarities and differences. Both models include source terms for: permitted wastewater discharge; area of developed land; nutrient mass in commercial fertilizer applied to various agricultural crops; and nutrient mass in manure from livestock, but other source terms reflect the differences in origin of the two nutrients. Most nitrogen is held in the earth's atmosphere while most phosphorus is held in or attached to soil particles (Hem, 1985). An atmospheric deposition source is thus applicable to the nitrogen model. Because of limitations in the explanatory variable data, however, it was applied in a way that represents regional sources (not local urban) and may not fully account for dry deposition contributions. The forested land source term, on the other hand, is unique to the phosphorus model.

Similarities and differences between the SPARROW models for nitrogen and phosphorus are also reflected in the land-to-water delivery terms. Both models demonstrate a negative relation between delivery of the nutrient and overland flow distance from the source to the stream channel. In both models, increased distance to the streams results in a reduced delivery of nutrients to the streams. In the phosphorus model an increase in groundwater flow is associated with a decreased delivery of phosphorus to the streams. This is consistent with current understanding of the dominant hydrologic pathways that affect nutrient mobility and delivery to streams (Nolan and Stoner, 2000): nitrogen moves readily through the groundwater system, whereas phosphorus does not because it tends to become attached to sediment particles. Temperature, as a land-to-water delivery term, was found to be significant only in the nitrogen model (and is inversely related to stream load, presumably because biologically mediated denitrification is limited by colder temperatures).

The land-to-water delivery term, natural log of the ratio of nitrate to TIN in wet deposition, is unique to the nitrogen model. The model shows that the delivery of atmospheric nitrogen to the streams increases as the proportion of nitrate in deposition increases. This finding is consistent with our knowledge that ammonium is strongly adsorbed on soil particles and is limited in its ability to move from the land to the streams, while nitrate is readily transported in water and is stable over a considerable range of conditions (Hem, 1985) and thus more apt to transport to the streams. Also, it is possible that nitrate, somewhere along the path to the stream, can be reduced to nitrite and react with the ammonium to produce nitrogen gas and water, and nitrogen gas could then be lost back to the atmosphere. Denitrification is recognized as an important component in the nitrogen cycle. Seitzinger *et al.* (2006) provides a good synopsis of the many complex processes involved in denitrification across the landscape and within the water network.

Aquatic decay terms also illustrate differences between SPARROW models for the two nutrients. In the nitrogen model, instream attenuation is identified as the only loss term, while for the phosphorus model nutrient loss is associated with lakes and reservoirs. The nitrogen model indicates that most of the removal occurs in small streams, those with mean-annual flows of 2.83 m^3^/s or less (100 ft^3^/s). An important characteristic of the nitrogen model is the lack of statistically significant nitrogen loss (on an annual basis) for large streams, those with flows greater than 2.83 m^3^/s and for reservoirs. The implication is that annual nitrogen loads that reach the larger rivers, or are discharged directly into these larger rivers, are also apt to travel to the estuary without any significant decay. We conclude that large rivers in the Northeast and Mid-Atlantic have a negligible rate of nitrogen attenuation on an annual basis, based on the estimated first-order reaction rate constants in our model and the uncertainties associated with the model coefficients. Our results are generally consistent with other modeling and field studies of denitrification (including other SPARROW models) that show that the first-order reaction rate constant declines with increases in stream depth, although other studies show small but detectable reaction rate constants for large rivers (Seitzinger *et al.*, 2002; Alexander *et al.*, 2000, 2008; Boyer *et al.*, 2006; Preston and Brakebill, 1999). The analysis by Seitzinger *et al.* (2002) also indicates that large rivers are capable of removing considerable absolute quantities of nitrogen despite their low reaction rate constants compared to those in small streams. The models presented here, showing a lack of or negligible attenuation, are consistent with the results of the New England SPARROW model (Moore *et al.*, 2004) which was tested and confirmed with additional field investigations, in which new data for the Connecticut River were collected and evaluated (Smith *et al.*, 2008). Possible reasons for differences in the magnitude of the reaction rates among studies might reflect differences in time and geographic extent as well as sensitivity of the measurement and modeling techniques. SPARROW models presented here for the NE US are based on mean annual estimates of nutrients and 2002 water-quality conditions. Many of the field studies are seasonal, with an emphasis on summer periods when conditions are well-suited for denitrification to occur (e.g., Alexander *et al.*, 2009). In any case, instream, lake, and reservoir nutrient attenuation is a topic ripe for further study, including the possibility of a reduction in attenuation rates over time due to the legacy of past and continued anthropomorphic nutrient loadings.

Sensitivity tests were conducted, using the calibrated NE US nitrogen SPARROW model, to see whether the model estimates of zero (i.e., negligible) nitrogen attenuation (on an annual basis) in the larger rivers is sensitive to selected predictor variables or to the restriction of the model estimates of instream attenuation to small streams. In the first test, we evaluated sensitivity to the estimates of the point sources (and possible errors in their reported values), given that many of the facilities discharge loads to large rivers. We found that a doubling (or halving) of all point source estimates simply halved (or doubled) the point source coefficient and resulted in absolutely no change in the stream decay and other coefficients. In the second test, we estimated a single stream decay coefficient for all streams. This caused the stream decay coefficient to drop from 0.224 (for small streams only) to a statistically insignificant (*p* = 0.58) value of 0.018 (for all streams). All other model coefficients in this test remained statistically significant with values similar to the original calibrated model coefficients. Other breakpoints for the small stream and large river classes were also evaluated but the breakpoint in the final model (mean-annual flows of 2.83 m^3^/s or less) gave the most statistically significant results. These tests suggest that the lack of (i.e., negligible) attenuation estimated for the large rivers is not an artifact of errors in the point source estimates nor related to the stream size restrictions on the estimated nitrogen decay coefficient.

The phosphorus model does not identify any significant instream attenuation for any stream class. Rather, the modeled phosphorus loss occurs in lakes and reservoirs. The two nutrients, nitrogen and phosphorus, are lost from the surface-water system in different ways. Nitrogen appears to be lost in the small streams via denitrification, where there is increased potential for exchange with the atmosphere, whereas phosphorus appears to be lost primarily in water bodies in which sediments settle out, presumably accumulating phosphorus attached to particles.

The nitrogen SPARROW model was found to be useful in examining the source and delivery of nitrogen to estuaries. This is important because nitrogen is often the limiting nutrient for aquatic plant growth in saltwater. The nitrogen SPARROW model indicates that agricultural sources contribute the largest percentage (37%) of the total combined nitrogen load delivered to the estuaries of nine large rivers within the region. The high agricultural source share for nitrogen is also dominated by the crop group of corn, soybean, and alfalfa that is grown there. Resource management goals to reduce nitrogen loads to the estuaries are thus apt to be in conflict with the trend to turn more agricultural land into growing these crops (as the demand for food and corn-based ethanol, a gasoline substitute and additive, increases).

One of the strengths of the SPARROW modeling technique is the ability to account for sources, transport, and fate of nutrients delivered to receiving waters. Unlike other management models that are tuned to reflect daily and seasonal conditions and processes, such as algal uptake, SPARROW is a long-term (mean annual) model and is thus useful in examining nutrient fate, including factors such as denitrification and long-term storage. This can be especially useful in water-resource management decisions. This information can be used to evaluate the relative importance of the various sources. For example, point sources are one of the more controllable sources and account for 28% of the nitrogen loads reaching the estuaries of the nine large rivers. As a result, a management decision could be made to go from secondary to tertiary treatment of point source discharges in order to remove much of the nitrogen prior to discharge. A previous SPARROW model for New England (Moore *et al.*, 2004) illustrated the utility of the model for the Providence River in Rhode Island and Massachusetts. The New England model predicted that 61% of the nitrogen load delivered by rivers to the estuary came from point sources. Narragansett Bay periodically experiences conditions of hypoxia especially at times of limited mixing of waters within the bay. SPARROW model results provide information that supports the decision to require the removal of nitrogen from upstream point sources in the Providence and Blackstone rivers watershed (Moore *et al.*, 2007). The nitrogen SPARROW model results (Table 3) indicate, as do previous models, that a number of the major rivers have estuaries with high point source shares of the total nitrogen load delivered to their estuaries. The nitrogen SPARROW model prediction compared well with results of the TMDL study that included the Connecticut River.

Atmospheric deposition of nitrogen is especially difficult to manage because it typically originates from beyond watershed boundaries and political jurisdictions. Complicating the management issue, SPARROW model results indicate that the form of the nitrogen makes a difference. The model shows that nitrate is much more effective in reaching the river system than ammonium. Atmospheric deposition accounts for 20% of the nitrogen loads reaching the estuaries of the nine large rivers. The importance of atmospheric deposition to nitrogen stream loads is in part dependent upon: the chemical form and deposition rate; the amount of loss in transit to the streams and loss within small streams; and the relative amount of other loads from the other sources including agricultural sources, developed lands, and point sources. In general, atmospheric deposition contributes an increasingly larger share of the nitrogen load as one proceeds northward within the NE US study area and inland toward the mountains. Atmospheric deposition is the largest source of nitrogen delivered to the estuaries for the watersheds of the Connecticut River, the Kennebec River and the Penobscot River, and comprises an especially high proportion of total nitrogen in the Kennebec and the Penobscot, where atmospheric deposition contributes more than half of the total delivered loads. Atmospheric deposition is a major source of nitrogen throughout the study area. Managing this nitrogen source is a major challenge, and under the Clean Air Act, requires the coordination between states and cooperation of multiple state agencies, federal agencies, industries, and other interested parties.

The phosphorus SPARROW model was used to examine the source and delivery of phosphorus to lakes/reservoirs. Phosphorus is the limiting nutrient for aquatic plant growth in freshwater lakes and ponds, and phosphorus accumulates in lakes and lake sediments. The SPARROW phosphorus model has the potential to be used to aid in the identification of lakes and lake embayments where accumulation is likely to occur more rapidly. Thirteen large lakes or reservoirs within NE US were selected and examined relative to SPARROW predictions for phosphorus sources, delivered loads, and accumulated loads. Results of the analysis showed a wide range of phosphorus accumulation within the lakes. The percent of the phosphorus load that accumulates within the selected lakes ranges from less than 1 to nearly 60% of the total load, depending upon the rate of hydraulic flushing in each reservoir. Reservoirs that are small relative to the size of their watershed (and streamflow), such as Cannonsville Reservoir and Lakes Clarke, Aldred, Conowingo, and Twin-Pemadumcook have low percentage losses. Greater flows lead to reduced residence time of water within a lake and less opportunity for loss to or accumulation of phosphorus in the lake. Model results for lakes that are large relative to the size of their watershed, such as Quabbin Reservoir, Lake Winnipesaukee, and Lake George, tend to have high percentages of phosphorus accumulation. Model results reflect the fact that phosphorus concentrations are greatest at or near the sites where streams first enter the lake or bay (Figure 7).

Integration of NLA (2007) lakes survey data with the SPARROW predictions built confidence in the SPARROW prediction results for lakes. The comparison of indirectly and independently predicted SPARROW phosphorus concentrations to concentrations measured in the NLA survey compared reasonably well, explaining 46 percent of the variance in the observed data. This reasonable comparison occurs despite the fact that SPARROW is a mean-annual stream model, developed separately from instantaneous lake concentration data. Mean-annual (long-term) flow-weighted lake concentrations are indirectly predicted by the SPARROW model.

SPARROW models are useful in identifying source shares of nitrogen and phosphorus loads to receiving waters (estuaries and lakes/reservoirs) throughout the NE US. Model results, available for each NHD flowline (Booth *et al.*, this issue), can be used to support TMDL applications where source shares and delivery of nutrients are of interest, such as those that were of concern in the Connecticut River–Long Island TMDL study (New York State Department of Environmental Conservation and Connecticut Department of Environmental Protection, 2000). Model results show that nutrient attenuation processes in smaller watersheds are especially important. The attenuation of nitrogen is greatest within the terrestrial environment before it ever reaches the stream network. Once the nitrogen enters the stream network its attenuation is found to be significant, on an annual basis, only in small streams with flows less than 2.83 m^3^/s; and even there attenuation is small (about 8% loss on average). For phosphorus, attenuation is greatest in lakes with small watersheds relative to the size of the lake. This finding has important management implications. The model results, in contrast to some studies and in agreement with others, indicate that aquatic decay of nutrients is quite limited on an annual basis and that we especially cannot rely on natural attenuation to remove nutrients within the larger rivers nor within lakes with large watersheds relative to the size of the lake.

## SUPPORTING INFORMATION

Additional supporting information may be found in the online version of this article. Included are: (A) discussion of the nitrogen model coefficients and the physical interpretation of these coefficients; (B) discussion of the nitrogen model results for 9 major watersheds and the relative role of atmospheric deposition within these watersheds; and (C) discussion of the phosphorus model coefficients and the physical interpretation of these coefficients.

Please note: Neither AWRA nor Wiley-Blackwell is responsible for the content or functionality of any supporting materials supplied by the authors. Any queries (other than missing material) should be directed to the corresponding author for the article.

## References

[b1] Alexander RB, Böhlke JK, Boyer EW, David MB, Harvey JW, Mulholland PJ, Seitzinger SP, Tobias CR, Tonitto C, Wollheim WM (2009). Dynamic Modeling of Nitrogen Losses in River Networks Unravels the Coupled Effects of Hydrological and Biogeochemical Processes. Biogeochemistry.

[b2] Alexander RB, Elliott AH, Shankar U, McBride GB (2002). Estimating the Sources and Transport of Nutrients in the Waikato River Basin, New Zealand. Water Resources Research.

[b3] Alexander RB, Smith RA, Schwarz GE (2000). Effect of Stream Channel Size on the Delivery of Nitrogen to the Gulf of Mexico. Nature.

[b4] Alexander RB, Smith RA, Schwarz GE, Boyer EW, Nolan JV, Brakebill JW (2008). Differences in Phosphorus and Nitrogen Delivery to the Gulf of Mexico From the Mississippi River Basin. Environmental Science & Technology.

[b5] Alexander RB, Smith RA, Schwarz GE, Preston SD, Brakebill JW, Srinivasan R, Pacheco PA, Valigura Richard, Alexander Richard, Castro Mark, Meyers Tilden, Paerl Hans, Stacey Paul, Eugene Turner R (2001). Atmospheric Nitrogen Flux From the Watersheds of Major Estuaries of the United States: An Application of the SPARROW Watershed Model. Nitrogen Loading in Coastal Water Bodies: An Atmospheric Perspective.

[b6] Booth NL, Everman EJ, Kuo I-L, Sprague L, Murphy L A Web-Based Decision Support System for Assessing Regional Water-Quality Conditions and Management Actions. Journal of the American Water Resources Association.

[b7] Boyer EW, Alexander RB, Parton WJ, Li CS, Butterbach-Bahl K, Donner SD, Skaggs RW, Del Grosso S (2006). Modeling Denitrification in Terrestrial and Aquatic Ecosystems at Regional Scales. Ecological Applications.

[b8] Boyer EW, Goodale CL, Jaworski NA, Howarth RW (2002). Effects of Anthropogenic Nitrogen Loading on Riverine Nitrogen Export in the Northeastern U.S.. Biogeochemistry.

[b9] Brakebill JW, Preston SD (2004). Digital Data Used to Relate Nutrient Inputs to Water Quality in the Chesapeake Bay Watershed, Version 3.0. http://md.water.usgs.gov/publications/ofr-2004-1433/.

[b10] Brakebill JW, Wolock DM, Terziotti SE Digital Hydrologic Networks Supporting Applications Related to Spatially Referenced Regression Modeling. Journal of the American Water Resources Association.

[b11] Bricker SB, Clement CG, Pirhalla DE, Orlando SP, Farrow DRG (1999). National Estuarine Eutrophication Assessment: Effects of Nutrient Enrichment in the Nation's Estuaries.

[b12] Bricker SB, Longstaff B, Dennison W, Jones A, Boicourt K, Wicks C, Woerner J (2007). Effects of Nutrient Enrichment In the Nation's Estuaries: A Decade of Change.

[b13] Brown JB, Sprague LA, Dupree JA Nutrient Sources and Transport in the Missouri River Basin, With Emphasis on the Effects of Irrigation and Reservoirs. Journal of the American Water Resources Association.

[b14] Fry JA, Coan MJ, Homer CG, Meyer DK, Wickham JD (2009). Completion of the National Land Cover Database (NLCD) 1992–2001 Land Cover Change Retrofit Product. http://pubs.usgs.gov/of/2008/1379.

[b15] García AM, Hoos AB, Terziotti S A Regional Modeling Framework of Phosphorus Sources and Transport in Streams of the Southeastern United States. Journal of the American Water Resources Association.

[b16] Gesch D, Evans G, Mauck J, Hutchinson J, Carswell WJ (2009). The National Map – Elevation. http://pubs.usgs.gov/fs/2009/3053/.

[b17] Helsel DR, Hirsch RM (1992). Statistical Methods in Water Resources.

[b18] Hem JD (1985). Study and Interpretation of the Chemical Characteristics of Natural Water.

[b19] Hoos AB, McMahon G (2009). Spatial Analysis of Instream Nitrogen Loads and Factors Controlling Nitrogen Delivery to Streams in the Southeastern United States Using Spatially Referenced Regression on Watershed Attributes. Journal of Hydrological Processes.

[b20] Howarth RW, Billen G, Swaney D, Townsend A, Jaworski N, Lajtha K, Downing JA, Elmgren R, Caraco N, Jordan T, Berendse F, Freney J, Kueyarov V, Murdoch P, Zhao-Liang Z (1996). Riverine Inputs of Nitrogen to the North Atlantic Ocean: Fluxes and Human Influences. Biogeochemistry.

[b21] Jaworksi NA, Howarth RW, Hetling LJ (1997). Atmospheric Deposition of Nitrogen Oxides onto the Landscape Contributes to Coastal Eutrophication in the Northeast US. Environmental Science and Technology.

[b22] Johnston CM, Dewald TG, Bondelid TR, Worstell BB, McKay LD, Rea A, Moore RB, Goodall JL (2009). Evaluation of Catchment Delineation Methods for the Medium-Resolution National Hydrography Dataset. http://pubs.usgs.gov/sir/2009/5233/.

[b23] Langland MJ (1998). Changes in Sediment and Nutrient Storage in Three Reservoirs in the Lower Susquehanna River Basin and Implications for the Chesapeake Bay.

[b24] Maupin MA, Ivahnenko T Nutrient Loadings to Streams of the Continental United States from Municipal and Industrial Effluent. Journal of the American Water Resources Association.

[b25] McMahon G, Tervelt L, Donehoo W (2007). Methods for Estimating Annual Wastewater Nutrient Loads in the Southeastern United States. http://pubs.usgs.gov/of/2007/1040/.

[b26] Moore RB, Johnston CM, Robinson KW, Deacon JR (2004). Estimation of Total Nitrogen and Phosphorus in New England Streams Using Spatially Referenced Regression Models. http://pubs.usgs.gov/sir/2004/5012/.

[b27] Moore RB, Walker HA, Dettmann EH (2007). Integration of National Coastal Assessment Data, Freshwater Nutrient (SPARROW) Modeling and Estuary Nutrient Mass Balance Calculations: An Example From Narragansett Bay. http://epa.gov/emap/html/pubs/docs/groupdocs/symposia/symp2007/abstracts/moore.html.

[b28] National Research Council (2000). Clean Coastal Waters Understanding and Reducing the Effects of Nutrient Pollution.

[b29] New York State Department of Environmental Conservation and Connecticut Department of Environmental Protection (2000). A Total Maximum Daily Load Analysis to Achieve Water Quality Standards for Dissolved Oxygen in Long Island Sound. http://www.ct.gov/dep/lib/dep/water/lis_water_quality/nitrogen_control_program/tmdl.pdf.

[b30] Nolan BT, Stoner JD (2000). Nutrients in Groundwaters of the Conterminous United States, 1992-1995. Environmental Science & Technology.

[b31] Olsen A, Snyder B, Stahl L, Pitt J (2009). Survey Design for Lakes and Reservoirs in the United States to Assess Contaminants in Fish Tissue. Environmental Monitoring and Assessment.

[b32] Omernik JM, Davis WS, Simon TP (1995). Ecoregions – A Framework for Environmental Management. Biological Assessment and Criteria-Tools for Water Resource Planning and Decision Making.

[b33] Preston SD, Alexander RB, Woodside MD, Hamilton PA (2009). SPARROW MODELING – Enhancing Understanding of the Nation's Water Quality. http://pubs.usgs.gov/fs/2009/3019/.

[b34] Preston SD, Brakebill JW (1999). Application of Spatially Referenced Regression Modeling for the Evaluation of Total Nitrogen Loading in the Chesapeake Bay Watershed. http://pubs.er.usgs.gov/usgspubs/wri/wri994054.

[b35] Rebich RA, Houston NA, Mize SV, Pearson DK, Ging PB, Hornig CE Sources and Delivery of Nutrients to the Northwestern Gulf of Mexico From Streams in the South-Central United States. Journal of the American Water Resources Association.

[b36] Robertson DM, Saad DA Nutrient Inputs to the Laurentian Great Lakes by Source and Watershed Estimated Using SPARROW Watershed Models. Journal of the American Water Resources Association.

[b37] Saad DA, Schwarz GE, Robertson DM, Booth NL A Multi-Agency Nutrient Dataset Used to Estimate Loads, Improve Monitoring Design, and Calibrate Regional Nutrient SPARROW Models. Journal of the American Water Resources Association.

[b38] SAS Institute, Inc. (1999). SAS User's Guide, version 8.

[b39] Schindler DW, Hecky RE, Findlay DL, Stainton MP, Parker BR, Paterson M, Beaty KG, Lyng M, Kasian SEM (2008). Eutrophication of Lakes Cannot be Controlled by Reducing Nitrogen Input: Results of a 37 Year Whole Ecosystem Experiment. Proceedings of the National Academy of Science USA.

[b40] Schwarz GE, Hoos AB, Alexander RB, Smith RA (2006). The SPARROW Surface Water-Quality Model: Theory, Application, and User Documentation. http://pubs.er.usgs.gov/usgspubs/tm/tm6B3.

[b41] Seitzinger S, Harrison JA, Bohlke JK, Bouwman AF, Lowrance R, Peterson B, Tobias C, Drecht GV (2006). Denitrification Across Landscapes and Waterscapes: A Synthesis. Ecological Applications.

[b42] Seitzinger SP, Styles RV, Boyer EW, Alexander RB, Billen G, Howarth RW, Mayer B, van Breemen N (2002). Nitrogen Retention in Rivers: Model Development and Application to Watersheds in the Northeastern U.S.A. Biogeochemistry.

[b43] Simley JD, Carswell WJ (2009). The National Map – Hydrography. http://pubs.usgs.gov/fs/2009/3054/.

[b44] Smith TE, Laursen AE, Deacon JR (2008). Nitrogen Attenuation in the Connecticut River, Northeastern USA; A Comparison of Mass Balance and N2 Production Modeling Approaches. Biogeochemistry.

[b45] Smith RA, Schwarz GE, Alexander RB (1997). Regional Interpretation of Water-Quality Monitoring Data. Water Resources Research.

[b46] Stevens DL, Olsen AR (2004). Spatially Balanced Sampling of Natural Resources. Journal of the American Statistical Association.

[b47] Strahler AN (1957). Quantitative Analysis of Watershed Geomorphology. Transactions of the American Geophysical Union.

[b48] U.S. Department of Agriculture, Natural Resources Conservation Service (2004). Watershed Boundary Dataset. http://www.ncgc.nrcs.usda.gov/products/datasets/watershed/.

[b49] U.S. Environmental Protection Agency (2000a). Nutrient Criteria Technical Guidance Manual: Office of Water.

[b50] U.S. Environmental Protection Agency (2000b). State of the Environment.

[b51] U.S. Environmental Protection Agency (2008). National Coastal Condition Report III. http://water.epa.gov/type/oceb/assessmonitor/downloads.cfm.

[b52] U.S. Environmental Protection Agency (2009). National Lakes Assessment: A Collaborative Survey of the Nation's Lakes.

[b53] U.S. Geological Survey (USGS) (2000). Atmospheric Deposition Program of the U.S. Geological Survey. http://bqs.usgs.gov/acidrain/Program.pdf.

[b54] Wieczorek ME, Lamotte AE (2011). Attributes for NHDPlus Catchments (Version 1.1) for the Conterminous United States. U.S. Geological Survey Digital Data Series DS-490. http://water.usgs.gov/nawqa/modeling/nhdplusattributes.html.

[b55] Wise DR, Johnson HM Surface-Water Nutrient Conditions and Sources in the United States Pacific Northwest. Journal of the American Water Resources Association.

[b56] Wolock DM (2003). Base-Flow Index Grid for the Conterminous United States. http://ks.water.usgs.gov/pubs/abstracts/of.03-263.htm.

