# Supplementary material for: Source and Delivery of Nutrients to Receiving Waters in the Northeastern and Mid-Atlantic Regions of the United States
Source: J Am Water Resour Assoc. 2011 Oct;47(5):965–90. doi: 10.1111/j.1752-1688.2011.00582.x (PMC3307627; doi:10.1111/j.1752-1688.2011.00582.x)
Supplement: Supplementary file 1 [file jawr0047-0965-SD1.pdf]

Supplement A – Nitrogen model coefficients

The coefficients estimated for the nitrogen model (Table 1) have various physical interpretations. Land-to-water variables are applied to each source term except for the point source which is discharged directly into the river, and the ratio of nitrate to total inorganic nitrogen which is applied to the atmospheric source. All values for the land-to-water variables were expressed as the difference from the mean value. This makes the coefficients for the source variables more interpretable and comparable from one model to another. The model has 6 source terms, 5 land-to-water delivery terms, and one in-stream attenuation term.

Examining the source terms, the source coefficient of 1.16 for discharges from municipal wastewater-treatment facilities indicates that for each estimated kilogram of nitrogen discharged into the rivers at the wastewater-discharge locations, the model predicts 1.16 (± 0.17) kg of nitrogen at the monitoring stations (minus any in-stream attenuation). A coefficient greater than one may be an indication that point source loads (input to SPARROW) are underestimated by about 16 %; however, the coefficient is well within the standard error from a value of one. Alternatively, there may be other unaccounted for sources that typically occur near point sources that also contribute nitrogen. The coefficient of 0.279 for atmospheric deposition indicates that for each estimated kilogram of nitrate that falls on the land surface, the model is predicting that an average of 0.279 (± 0.028) kg of nitrogen are delivered to streams. In other words, the results of the model indicate that a large percentage of the mass of nitrogen from atmospheric deposition is either retained in the watershed or eventually returned to the atmosphere.

The coefficient for developed lands indicate that about 1,422 (±169) kg of nitrogen occur in streams for each square kilometer (kg/ km^2) of developed land upstream per year. This is considerably higher than the median export coefficient for nitrogen for urban lands of 550 kg/km^2 reported by Reckhow *et al.*, (1980) for a variety of watersheds across the U.S. and Canada. It is however within the range of reported export coefficients (Reckhow *et al.*, 1980) which have a mean of 997 kg/km^2 and a maximum value of 3,847 kg/km^2.

There are three agricultural source terms for nitrogen in the model. The first, nitrogen mass in commercial fertilizer applied to, and fixation from, agricultural land in corn, soybean, or alfalfa, has a coefficient of 0.310. This implies that about 31 % of the nitrogen from this source reaches the stream network. Corn, soybean, and alfalfa are typically planted in rotation to take advantage of fixation associated with soybean and especially alfalfa. For these crops it is difficult to separate out the effects of fertilizer and fixation. As a result of this uncertainty in interpretation, model input, estimates of nitrogen from corn, soybean, and alfalfa are lumped (summed) into just one category associated with the crop group of corn, soybean, and alfalfa. A second source from agriculture is nitrogen mass in commercial fertilizer applied to agricultural land in crops other than corn, soybean, alfalfa, wheat, or hay. The coefficient for this source (0.186) indicates that about 19 % of the nitrogen from this source reaches the stream network. The fourth and final agricultural source is nitrogen mass in manure from livestock production. The coefficient for manure (0.090) indicates that about 9 % of the nitrogen from manure reaches the stream network.

For land-to-water delivery terms, the coefficients influence stream loads in the following ways. The negative coefficient (-0.864) associated with mean annual temperature means that increases in mean

temperature retards the delivery of nutrients to the streams, perhaps indicating increased consumption of nitrogen by plants with warmer temperatures. The negative coefficient (-0.190) associated with overland flow distance to the stream channel means that increased distance to the streams retards the delivery of nutrients to the streams. A positive coefficient (2.56) associated with the variable natural log of the ratio of nitrate to total inorganic nitrogen in wet nitrogen deposition indicates that delivery of nitrogen to the streams increases as the proportion of nitrate in the wet atmospheric deposition increases. This reflects the greater mobility of nitrate compared to other forms of nitrogen. This relation applies only to the atmospheric nitrogen source and how readily atmospheric deposited nitrogen reaches the streams. Positive coefficients for two of the Ecoregions, the Northern Piedmont Ecoregion (0.422) and Valley and Ridge Ecoregion (0.593), indicates that delivery to the streams is enhanced within these two ecoregions. A possible explanation for this may relate to the fact that these ecoregions have carbonate soils and carbonate bedrock associated with them (Woods *et al.*, 1996). Dissolution of carbonate soils and rock may result in enhanced delivery of the nitrogen through the groundwater within these ecoregions.

In-stream attenuation is significant in the model for only one stream class. Decreasing nitrogen attenuation with increasing stream size has been noted by numerous studies (Smith *et al.*, 1997, Preston and Brakebill, 1999, Alexander, *et al.*, 2000, and Moore *et al.*, 2004). A coefficient applies to streams, with mean-annual flows of 2.83 m^3/s or less (100 ft^3/s). The model indicates that the greatest rate of nitrogen removal occurs in these small streams. About 89 % of the stream reaches in the NE US model have mean-annual flows less than or equal to 2.83 m^3/s. Presumably, the ability to estimate loss in small streams was enhanced by using the detailed NHD stream network and monitoring stations on small drainages. The modeled loss thus applies to about 89 % of the stream reaches in the model. An important result was a lack of statistically significant nitrogen loss for large streams with flows greater than 2.83 m^3/s and for reservoirs. The implication of this is that annual nitrogen loads that make it into the medium sized and larger rivers, or are discharged directly into these larger rivers, are also apt to make it all the way to the estuary without any significant decay.

Literature Cited

Alexander, R.B., and R.A. Smith, and G.E. Schwarz, 2000, Effect of stream channel size on the delivery of nitrogen to the Gulf of Mexico, *Nature*, 403, p. 758-761.

Moore, R.B., C.M. Johnston, K.W. Robinson, and J.R. Deacon, 2004, Estimation of total nitrogen and phosphorus in New England streams using spatially referenced regression models: U.S. Geological Survey Scientific Investigations Report 2004–5012, 50 p., available online at http://pubs.usgs.gov/sir/2004/5012/.

Preston, S.D., and J.W. Brakebill, 1999, Application of spatially referenced regression modeling for the evaluation of total nitrogen loading in the Chesapeake Bay watershed: U.S. Geological Survey Water-Resources Investigations Report 99–4054, 12 p., http://pubs.er.usgs.gov/usgspubs/wri/wri994054 .

Reckhow, K.H., M.N. Beaulac, and J.T. Simpson, (1980). “Modeling phosphorus loading and lake response under uncertainty: A manual and compilation of export coefficients,” U.S. EPA Report No. EPA-440/5-80-011, Office of Water Regulations, Criteria and Standards Division, U.S. Environmental Protection Agency, Washington, DC.

Smith, R.A., G.E. Schwarz, and R.B. Alexander, 1997, Regional interpretation of water-quality monitoring data: Water Resources Research, v. 33, p. 2781–2798, available online at http://water.usgs.gov/nawqa/sparrow/wrr97/results.html

Woods, A.J., J.M. Omernik, D.D. Brown, and C.W. Kiilsgaard. 1996. Level III and IV ecoregions of Pennsylvania and the Blue Ridge Mountains, the Ridge and Valley, and Central Appalachians of Virginia, West Virginia, and Maryland. EPA/600/R-96/077. U.S. Environmental Protection Agency, National Health and Environmental Effects Research Laboratory, Corvallis, OR. 60p.

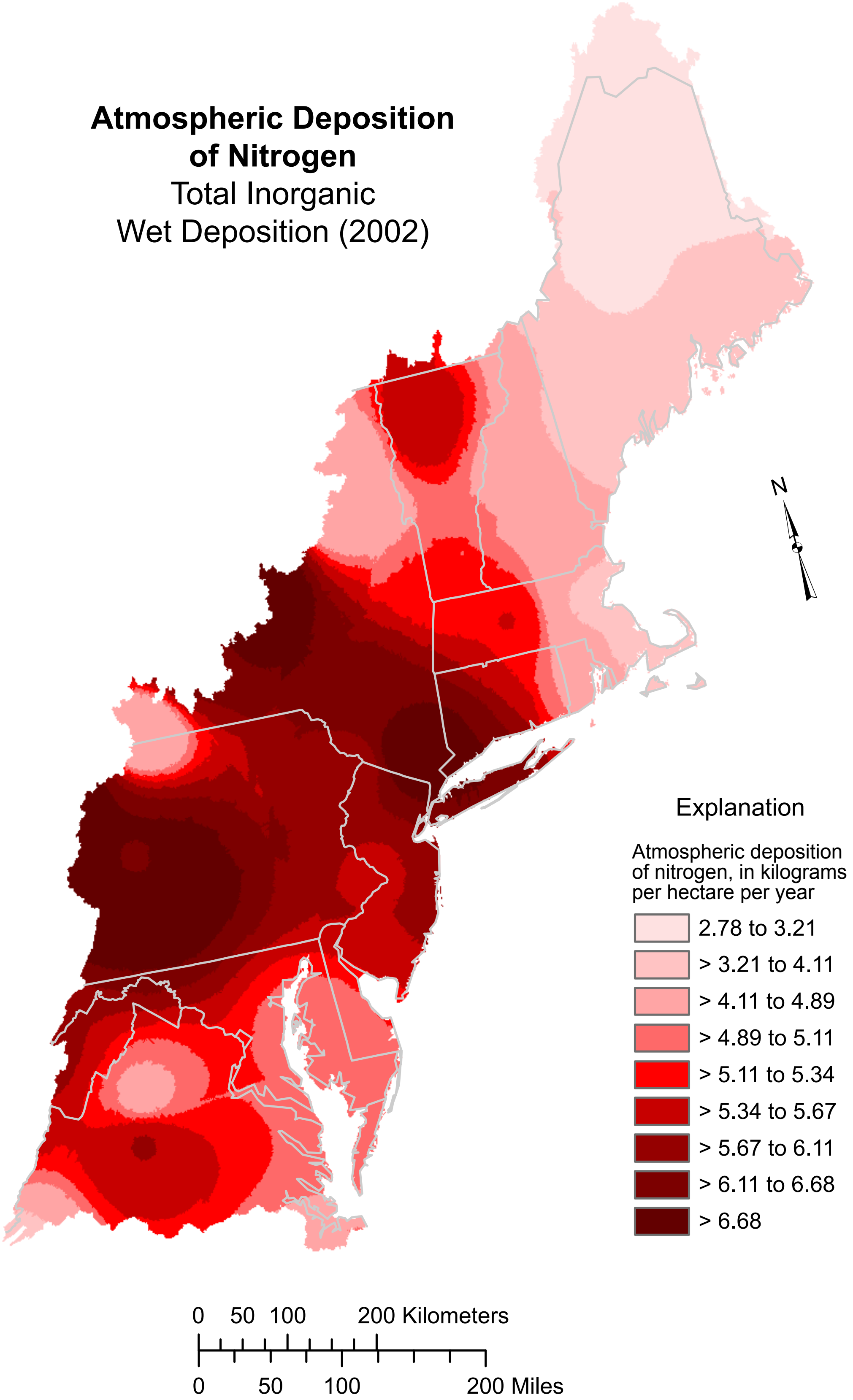

Figure S1. Atmospheric Deposition of Nitrogen within the Northeastern and Mid-Atlantic regions study area.

Supplement B – Nitrogen model results by major river – the relative role of atmospheric deposition

Atmospheric deposition of nitrogen is known to be a major source of nitrogen to streams (Smith *et al.*, 1997; Valigura *et al.*, 2001). The effect of atmospheric deposition of nitrogen is of special interest to water-quality managers because it often originates beyond the watershed and may be the largest source of nitrogen in some watersheds. Unlike other sources that are governed by the Clean Water Act, the federal legislation that applies to atmospheric nitrogen deposition is the Clean Air Act. Entirely different management teams are involved with monitoring and managing this source of nitrogen pollution. Figure S1 illustrates the detrended 2002 atmospheric deposition of nitrogen (Alexander *et al.*, 2001) used as input to the model. The relative contribution of atmospheric deposition to each estuary is more complicated than this map illustrates. The atmospheric sources modeled represent regional sources (not local sources) of wet deposition. The modeled data does not account for dry deposition contributions. Also, developed land likely includes N deposition contributions from local vehicle emissions. Additionally, atmospheric nitrogen decays by both land-to-water delivery processes and by instream losses. Furthermore, the relative role of atmospheric deposition of nitrogen increases as other sources decline. For example, the northeastern most part of the study area has some of the lowest deposition rates yet the importance of this source is relatively high because of a decrease in other sources.

Results indicate that atmospheric deposition of nitrogen affects the James River watershed (15.7% source share) less than most other watersheds. Model allocations also indicate that about half (50.1%) of the loads are from point sources, while agricultural sources account for 20% of the load. Agricultural sources are split mainly between manure applications and sources associated specifically with the crop group corn, soybean, and alfalfa (crops which are apt to be grown in rotation).

Loads reaching the Potomac River estuary come from 4 states and from the District of Columbia. Source allocations to the Potomac River estuary vary considerably within these political areas. Atmospheric deposition is not the primary source for any of the states but its contribution varies considerably, comprising as much as 32.3 % of West Virginia's share load down to only 0.3 % of the source load for the District of Columbia. The source share of atmospheric deposition is greatest in the western less populated mountainous part of the watershed. Point source loads dominate the District of Columbia with 97.5% of the load coming from point sources. Agricultural source shares for the 4 states range from 42.0% for Virginia to 71.2% for Pennsylvania. The crop group of corn, soybean, and alfalfa dominate in the Pennsylvania and Maryland portion, while elsewhere the agricultural source share are more evenly split between the crop group of corn, soybean, and alfalfa source and the manure source.

For the Susquehanna River, the modeled loads reaching the mouth of the Susquehanna River come from 3 states, Pennsylvania, New York, and Maryland. The State of Pennsylvania has the largest modeled allocated source accounting for about 83% of the total. New York accounted for 16% of the loads delivered to the mouth of the Susquehanna River and Maryland accounted for 1%. The two states contributing the most significant loads, Pennsylvania and New York have similar source allocations for all sources. Agricultural sources dominate both states' portion of the Susquehanna watershed with 60.3% of Pennsylvania's source share and 52.5% New York's source share. The crop group of corn, soy bean, and alfalfa is the dominate source for both states, with these crops accounting for about 39.9 % and 34.2 % respectively of nitrogen loads reaching the mouth of the Susquehanna River.

Point sources dominate the Delaware River watershed and account for about 46 % of the total nitrogen load reaching the Delaware River estuary. The loads reaching the Delaware River estuary come from 5 states, Pennsylvania, New Jersey, New York, Delaware, and Maryland, although the Maryland load contributions are negligible. Source allocations to the Delaware River estuary vary considerably within these political areas. Atmospheric deposition is the primary source for the New York portion at the northern end of the watershed with 55.2% of the source share. Point sources are the dominant source for the Pennsylvania, and New Jersey parts of the watershed, while point sources are approximately equal to agricultural sources for the Delaware part of the watershed. Agricultural sources have the second largest source share in the watershed. Excluding the small area in Maryland, the agricultural source share by state is remarkably uniform, ranging from 23.6% for New York to 36.4% for Delaware. The crop group of corn, soy bean, and alfalfa dominate the agricultural loads from all 5 states.

The State of New York contributes most (86%) of the estimated loads reaching the estuary of the Hudson River. In New York the primary sources are agricultural (31.3%) and point sources (28.7%), and atmospheric deposition, which comprises a large part of the New York source share (25.3%). The New Jersey part of the watershed, on the other hand, is almost entirely dominated by point sources contributing an estimated 85.9% of New Jersey's nitrogen loads to the Hudson River estuary. This is because of the dense population in the New Jersey part of the Hudson River watershed.

Nitrogen loadings to Long Island Sound from the Connecticut River come from 5 states, Massachusetts, Connecticut, Vermont, New Hampshire, and Maine, as well as from a small area within the Quebec Province of Canada. The Connecticut River watershed is the first example given where atmospheric deposition is the largest source reaching the estuary for the entire watershed (33.5% of the delivered source share). The relative contribution of atmospheric deposition does, however, vary by state. In the less populous northern states of Vermont and New Hampshire, the atmospheric source shares are at 54.0% and 55.8% respectively. In addition to fewer people in these states, the northern part of the watershed is more mountainous and receives more precipitation and more deposition of atmospheric nitrogen. Further south in the watershed, in the states of Massachusetts and Connecticut, other sources become more important. Point sources are the dominant source delivered to the estuary from both Massachusetts (33.2%) and Connecticut (54.7% delivered source share). Source allocations, among the agricultural sources, are much more varied than in the more southern watersheds and are not dominated by the crop group of corn, soy bean, and alfalfa.

In the Northern New England States of New Hampshire and Maine atmospheric deposition dominates the nitrogen source loads. Nitrogen loadings to the Merrimack River estuary, for example, from New Hampshire deliver 38.4% of that state's source share to the estuary. In the Massachusetts part of the Merrimack watershed, however, the point sources dominate (54.3%). For the Kennebec River watershed of Maine and New Hampshire, atmospheric deposition dominates, providing an estimated 54% of the total nitrogen loads reaching the estuary. Much of the basin is mountainous and not farmed, so agricultural sources from New Hampshire are very small (1.6%) and only moderate in Maine (12.7%). The Penobscot River watershed is located entirely within the State of Maine, and there again atmospheric deposition dominates by providing 66.4% of the nitrogen loadings to the Penobscot River Estuary.

Literature Cited

Alexander, R.B., R.A. Smith, G.E. Schwarz, S.D. Preston, J.W. Brakebill, R. Srinivasan, and P.A. Pacheco, 2001, Atmospheric Nitrogen Flux from the Watersheds of Major Estuaries of the United States: An Application of the SPARROW Watershed Model, In: *Nitrogen Loading in Coastal Water Bodies: An Atmospheric Perspective,* American Geophysical Union Monograph 57, Valigura, R.A., Alexander, R.B., Castro, M.S., Meyers, T.P., Paeri, H.W., Stacey, P.E., and Turner, R.E. [Eds], pp. 119-170.

Smith, R.A., G.E. Schwarz, and R.B. Alexander, 1997, Regional interpretation of water-quality monitoring data: *Water Resources Research*, v. 33, p. 2781–2798, available online at http://water.usgs.gov/nawqa/sparrow/wrr97/results.html

Valigura, R.A., R.B. Alexander, M.S. Castro, T.P. Meyers, H.W. Paeri, P.E. Stacey, and R.E. Turner, [Eds], 2001. Nitrogen loading in coastal water bodies: an atmospheric perspective. Coastal and Estuarine studies. American Geophysical Union Monograph 57, American Geophysical Union, Washington, DC, 254 p

Supplement C – Phosphorus model coefficients

The coefficients estimated for the phosphorus model (Table 1) have various physical interpretations. The model has 6 source terms, 4 land-to-water delivery terms, and 1 lake/reservoir loss term. Examining the source terms, the source coefficient of 1.32 for discharges from municipal wastewater-treatment facilities indicates that for each estimated kilogram of phosphorus discharged into the rivers at the wastewater-discharge locations, the model predicts 1.32 (± 0.22) kg of phosphorus at the monitoring stations (minus any in-stream attenuation). A coefficient greater than one may be an indication that point source loads (input to SPARROW) are underestimated by about 32 %. Alternatively, there may be other unaccounted for sources that typically occur near point sources that also contribute phosphorus. The coefficient for developed lands indicate that 106.3 (±14.2) kg of phosphorus reaches the stream network for each square kilometer of developed land upstream per year. This compares closely to a median phosphorus export coefficient for urban lands of 110 kg/km^2 reported by Reckhow *et al.*, (1980) for a variety of watersheds across the U.S. and Canada. There are three agricultural source terms for phosphorus in the model, all of which have coefficients that suggest that only a fraction of the source loading is delivered to streams. The first, phosphorus mass in commercial fertilizer applied to agricultural land in corn, soybean, and alfalfa, has a coefficient of 0.07. This implies that about 7 % of phosphorus fertilizer applied to agricultural land in corn, soybean, and alfalfa, the model reaches the stream network. The second source from agriculture is phosphorus mass in commercial fertilizer applied to agricultural land in crops other than corn, soybean, alfalfa, wheat, or hay. This source has a coefficient of 0.23, which implies that about 23 % of the phosphorus in fertilizer applied to other crops the reaches the stream. The third and final agricultural source is phosphorus mass in manure from livestock production. The manure source has a coefficient of 0.056, which implies that about 6 % of the phosphorus in manure reaches the stream. The coefficient for forested lands indicate that about 11.4 (±1.7) kg of phosphorus reaches the stream network for each square kilometer of developed land upstream per year. This compares to the median phosphorus export coefficient for forested lands of 20.6 kg/km^2 reported by Reckhow *et al.,* (1980).

For land-to-water delivery terms the coefficients influence stream loads in the following ways. The negative coefficient (-0.996), applies to the variable that defines the percentage of streamflow coming from groundwater. It can be interpreted as meaning that an increased groundwater flow is associated with decreased delivery of phosphorus to the streams (i.e. the groundwater system is more effective in removing phosphgorus). This is the opposite of the nitrogen model, implying that the two nutrients are transported by very different mechanisms. Nitrogen tends to move readily with groundwater while phosphorus does not. This is consistent with the observation that phosphorus tends to be attached to and transported with sediment particles carried by the stream (Hem, 1985). The negative coefficient (-0.58) associated with overland flow distance to the stream channel means that increased distance to the streams retards the delivery of nutrients to the streams. A positive coefficient for the Eastern Great Lakes and Hudson Lowlands Ecoregion (0.97), indicates that delivery to the streams is enhanced within this ecoregion. The The Hudson Lowlands are associated with agricultural activity (U.S. Environmental Protection Agency, 1996) and may have an enhanced surface water component of stream flow as well as more phosphorus in the environment due to agriculture. Conversely the negative coefficient (-0.543) associated with the Northeastern Coastal Zone Ecoregion indicates that delivery to the streams in that ecoregion is attenuated. This ecoregion is associated with nutrient poor soils (U.S. Environmental Protection Agency, 1996) which may contribute to the phosphorus attenuation.

The lake/reservoir loss term has a coefficient of 2.69 meters per year. The physical meaning of this term, applied to all lakes and reservoirs, is that the phosphorus loss per unit surface area, of a part of a lake or reservoir that is associated with an artificial path within that waterbody, is directly proportional to the SPARROW-predicted concentration for each associated artificial path. The lack of an in-stream attenuation term and presence of a lake/reservoir loss term is again just the opposite of the nitrogen model. This again implies that the two nutrients are transported and lost in different ways from one another.

Literature Cited

Hem, J.D., 1985, Study and interpretation of the chemical characteristics of natural water: U.S. Geological Survey Water-Supply Paper 2254, 3rd ed., 263 p.

Reckhow, K.H., M.N. Beaulac, and J.T. Simpson, 1980, "Modeling phosphorus loading and lake response under uncertainty: A manual and compilation of export coefficients," U.S. EPA Report No. EPA-440/5-80-011, Office of Water Regulations, Criteria and Standards Division, U.S. Environmental Protection Agency, Washington, DC

U.S. Environmental Protection Agency, 1996, Level III ecoregions of the continental United States (revision of Omernik, 1987): Corvallis, Oregon: U.S. Environmental Protection Agency - National Health and Environmental Effects Research Laboratory Map M-1, various scales.
